# Observation of angular momentum transfer among crystal lattice modes

**DOI:** 10.1038/s41567-026-03274-8

**Published:** 2026-05-12

**Authors:** Olga Minakova, Carolina Paiva, Maximilian Frenzel, Michael S. Spencer, Joanna M. Urban, Christoph Ringkamp, Martin Wolf, Gregor Mussler, Dominik M. Juraschek, Sebastian F. Maehrlein

**Affiliations:** 1 Department of Physical Chemistry, Fritz Haber Institute of the Max Planck Society, Berlin, Germany; 2 School of Physics and Astronomy, Tel Aviv University, Tel Aviv, Israel; 3 Peter Grünberg Institut (PGI-9), Forschungszentrum Jülich, Jülich, Germany; 4 JARA-Fundamentals of Future Information Technology, Jülich-Aachen Research Alliance, Forschungszentrum Jülich GmbH and RWTH Aachen University, Jülich, Germany; 5 Department of Applied Physics and Science Education, Eindhoven University of Technology, Eindhoven, Netherlands; 6 Institute of Radiation Physics, Helmholtz-Zentrum Dresden-Rossendorf, Dresden, Germany; 7 Institute of Applied Physics, Dresden University of Technology, Dresden, Germany

**Keywords:** Condensed-matter physics, Nonlinear phenomena, Terahertz optics

## Abstract

Transfer of energy and linear momentum between lattice vibrations via anharmonic coupling is an important concept in solid-state physics. However, it remained difficult to directly observe how angular momentum is exchanged and conserved among lattice modes, even though these processes are thought to play an important role in achieving magnetization equilibrium and in spin relaxation effects like the Einstein–de Haas effect. Here we demonstrate and coherently control angular momentum transfer between two lattice modes using the inverse process of anharmonic decay. The observed rotational phonon–phonon Umklapp scattering enforces the conservation of quantized crystal angular momentum, as dictated by the discrete rotational symmetry of the crystal. We thereby experimentally confirm the fundamental analogy between linear and angular momentum conservation in solids. Moreover, we establish axial nonlinear phononics as a promising handle for the ultrafast control of material properties.

## Abstract (web summary)

How angular momentum is exchanged and conserved among lattice modes has been difficult to measure experimentally, but has now been observed via a coherent three-phonon scattering process in a topological insulator.

## Main

Fundamental physics is dictated by symmetry principles, linking conservation laws to time, space translation and rotational invariance. In crystalline solids, discrete translational symmetry enforces the conservation of pseudolinear momentum, also called crystal momentum, forming the basis of Bloch’s theorem, band structure theory and modern semiconductor-based technology. Therefore, quantized vibrations of the crystalline lattice (phonons) must conserve energy and linear crystal momentum during their interactions, which are only permitted by anharmonic phonon–phonon scattering requiring at least a three-phonon interaction. Such energy and momentum relaxation through anharmonic phonon–phonon interaction is fundamentally required to establish equilibrium states of condensed matter. The observation of phonon Umklapp scattering processes, for example, governing thermal conductivity, serves as a clear signature of conserved linear crystal momentum imposed by the periodicity of the lattice^1^.

However, the conservation and redistribution of angular momentum within the lattice, fundamentally required to reach magnetization equilibrium^2–4^ and imperative for spin relaxation phenomena in solids, remains a postulate^5,6^. Since the discovery of the Einstein–de Haas effect^2^, it has been recognized that electron spin angular momentum must eventually transfer to the lattice as a rigid-body rotation, bridging quantum spin dynamics with classical mechanical angular momentum^3,7^. Yet, how this transfer proceeds after the initial spin-lattice coupling^4,8–10^ and how it relates to the discrete rotational invariance of the lattice is still a black box so far. In contrast to the continuous rotational invariance of free space, in solids, a discrete *n*-fold rotational invariance *C*_*n*_ can only conserve angular momentum modulo *n* in the form of ‘pseudo’—here also called ‘crystal’—angular momentum. Yet, the direct experimental observation of phonon trajectories supporting this conceptual analogy between linear and angular momentum conservation in solids has remained elusive. Recently, lattice angular momentum in the form of circularly polarized chiral^11–13^ or axial^14^ phonons has been linked to thermal Hall conductivity^15^, ultrafast demagnetization^4,8^, and transient magnetic fields^16–19^ harnessed for magnetic switching^20^ or dynamical multiferroicity^21,22^. Nevertheless, the corresponding phonon trajectories, which would provide a direct observation of phonon angular momentum, could not be assessed in these studies, and the magnitude of their contribution to the observed magnetic effects remains debated^23–25^.

To prepare a net angular momentum by the circular polarization of a doubly degenerate phonon mode, its orthogonal components need to be coherently driven with a phase delay of π/2. For infrared (IR)-active E_u_ modes, this can be achieved by a circularly polarized laser pulse in the terahertz (THz) spectral domain, where the resonant (linear) coupling between the THz electric field and the phonon’s electric dipole directly imprints the helicity of light onto the phonon mode. Yet, the resulting phonon angular momentum has only been inferred from secondary effects, such as phonon magnetic fields^19,20,22^ or THz field-induced second harmonic generation^18^. For Raman-active modes, the lattice trajectory can be controlled and directly observed by all-optical experiments that utilize stimulated Raman scattering techniques^26^. In all these examples, angular momentum was transferred from the light field to the lattice mode, and then potentially further to spin states, which are delicate to infer from magneto-optic measurements^23,25^. However, tracing the consecutive flow of angular momentum from the initially excited phonon to other lattice modes has remained elusive and represents a huge gap in our understanding of the ultrafast demagnetization sequence and other spin-lattice-coupled phenomena since the pioneering experiments by Einstein, de Haas^2^ and Barnett^27^.

## Experimental concept

**Fig. 1:**
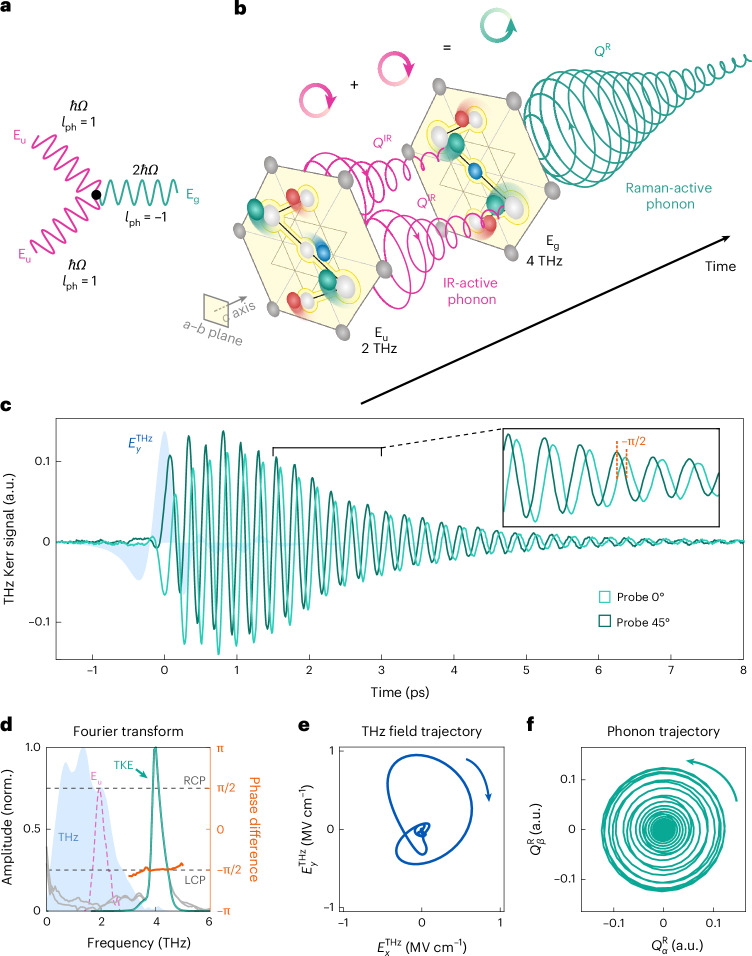
Phonon–phonon angular momentum transfer in bismuth selenide. **a**, Investigated three-phonon scattering process: annihilation of two E_u_ phonons producing one E_g_ phonon must conserve energy, (pseudo)linear momentum and angular momentum. **b**, Experimental concept: a coherent circular E_u_ mode (pink) at 2 THz nonlinearly transfers energy and momenta to the circular E_g_ mode (green) at 4 THz with opposite helicity in Bi_2_Se_3_. **c**, TKE in Bi_2_Se_3_ (green) under right-handed elliptical THz excitation (blue projection) for two linear probe pulse polarizations (0° and 45°) measuring two orthogonal E_g_ phonon components. Inset: zoomed-in view of the time-domain traces. **d**, Fourier transforms unveil equal amplitudes (green), but perfect −π/2 phase difference (orange) of the E_g_ phonon projections in **c**, clearly outside the excitation spectrum (blue shade) and at twice the E_u_ phonon resonance (pink). **e**, Trajectory of the THz excitation pulse’s electric field vector. **f**, E_g_ phonon trajectory obtained by Fourier-filtered TKE signal from **c** unveils opposite helicity with respect to the THz field in **a**. Source data

**Extended Data Fig. 1:**
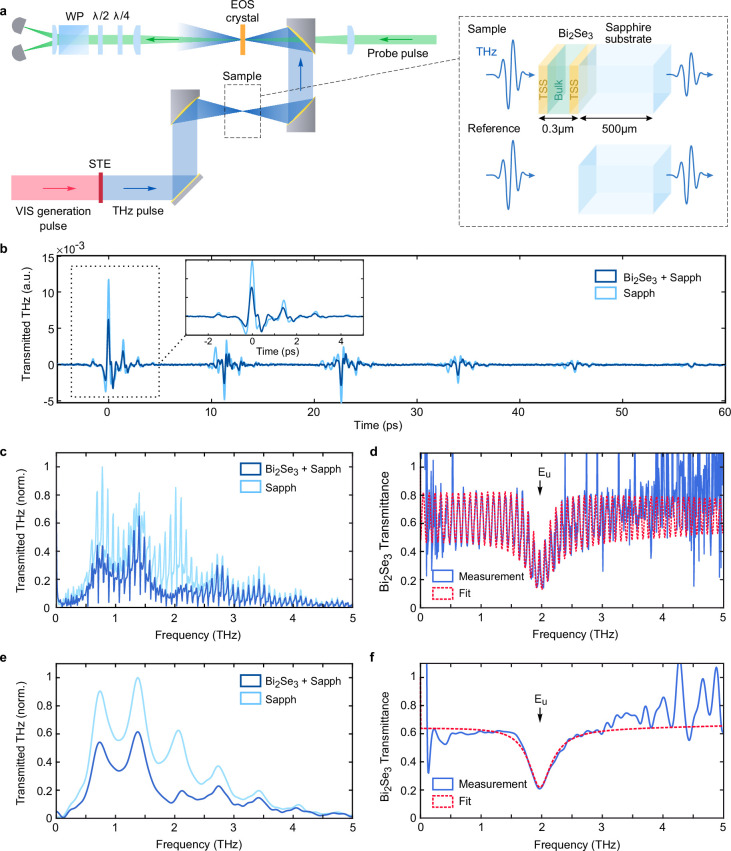
Terahertz time-domain spectroscopy in Bi_2_Se_3_. **a**, Schematic of the THz transmission setup. An ultrabroadband THz pulse from a large-area spintronic emitter is focused onto the sample position at the first focal point. The transmitted field is then refocused onto a 30 *μ*m-thick GaP crystal for electro-optic detection. The sample position hosts either a 0.3 *μ*m-thick Bi_2_Se_3_ film on a 500 *μ*m sapphire substrate or a bare 500 *μ*m-thick sapphire wafer for reference measurement. **b**-**c**, Time-domain signals of the sample (dark blue) and reference (light blue) THz transmission measurement, including their corresponding Fourier transforms. **d**, Division of the THz spectrum transmitted through the sample (panel **c**, dark blue) by the reference spectrum (panel **c**, light blue), revealing the THz transmittance of Bi_2_Se_3_ and the absorption feature of the E_u_ phonon mode. The modeled Bi_2_Se_3_ transmittance function (red line) shows very good agreement. **e**, Fourier transforms of the fundamental transmitted pulse obtained by applying a 8 ps-time window to **b** (see inset). **f**, Corresponding ratio of transmitted THz spectra, as in **d**. Isolation of the fundamental transmitted pulses removes Fabry-Pérot oscillations from the transmission spectrum, enabling more precise modeling of the E_u_ phonon feature. Source data

Here we provide experimental evidence for angular momentum *l*_ph_ transfer between lattice modes by a coherent three-phonon scattering process (Fig. 1a). As an archetypal example, we use ionic Raman scattering, which is permitted by the lowest-order anharmonic lattice potential that couples two quanta of IR-active phonons to a Raman-active phonon. The topological insulator Bi_2_Se_3_ provides an ideal material platform to coherently drive such a three-phonon scattering process of axial phonons: due to its inversion symmetry, Bi_2_Se_3_ accommodates either purely Raman- or IR-active doubly degenerate phonons in the *a*–*b* plane parallel to the surface. At room temperature, an IR-active E_u_ mode at 2.0 THz dominates the THz absorption^28^ (Extended Data Fig. 1). A Raman-active E_g_ mode at 4.0 THz is anharmonically coupled to this 2-THz mode^29^, perfectly fulfilling the double-frequency resonance condition 2*Ω*^IR^ = *Ω*^R^ (Methods) for sum-frequency ionic Raman scattering^30,31^.

As shown in the experimental concept (Fig. 1b), we first resonantly excite a circularly polarized E_u_ mode. In contrast to recent pioneering works^16,18,20,22^ using such axial IR phonons, we are not looking at the magneto-optic effects to study the angular momentum transfer to the spin system; instead, we follow the trajectory of the coupled E_g_ mode via the non-magnetic THz-induced Kerr effect (TKE)^32–34^. As both conservation of energy 2*ℏΩ*^IR^ = *ℏΩ*^R^ and linear momentum 2*ℏk*^IR^ = *ℏk*^R^ ≈ 0 (all Γ modes) are perfectly fulfilled in this nonlinear process, we can study the isolated dynamics of angular momentum transfer and its conservation. This coherent upconversion-type coupling, thus, serves as a test bed to observe the fundamental mechanism of phonon–phonon angular momentum transfer, which can be generalized to any symmetry-allowed higher linear momentum, higher-order or incoherent multiphonon scattering processes^1,35^. With this experimental design, we witness a helicity switch from one mode to the other, corresponding to the conservation of crystal angular momentum by a phononic analogue to a rotational Umklapp process^36,37^.

## Preparation and observation of coherent phonon angular momentum states

**Extended Data Fig. 2:**
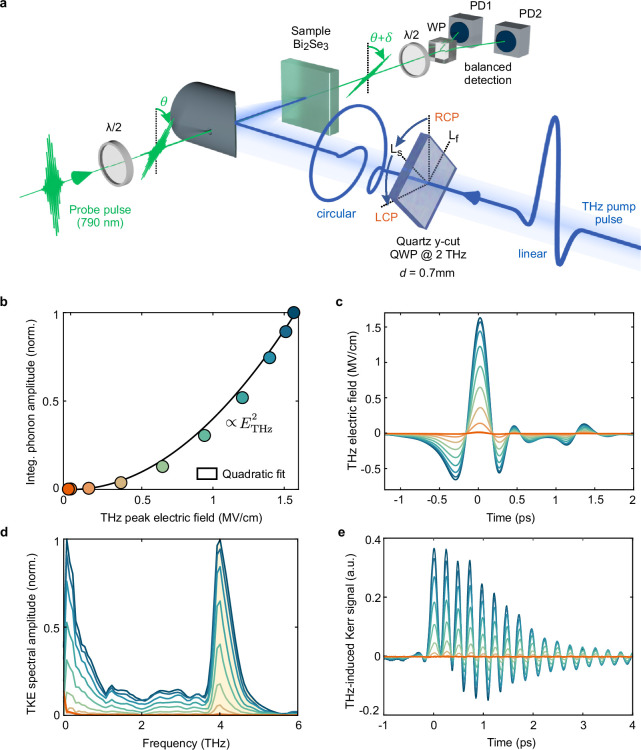
Experimental TKE setup and THz field dependence. **a**, Schematic of the experimental TKE setup. A THz pulse (blue), generated via optical rectification in LiNbO_3_, passes through a 0.7 mm-thick y-cut quartz waveplate to acquire a defined ellipticity state before irradiating the sample at normal incidence. The excited Raman-active phonon is detected via transient birefringence, which induces a time-dependent rotation of the probe pulse (green) polarization measured by a balanced detection. The angle of the incident probe pulse’s linear polarization is controlled by a half-wave plate. **b**-**e**, THz fluence dependence of the Raman-active E_g_ phonon. **b**, Integrated spectral amplitudes of the E_g_ mode as a function of THz peak electric field (coloured points) with a corresponding quadratic fit (black). Colours denote different THz peak electric fields and are used consistently across panels **b**-**e**. **c**, Incident THz fields measured by electro-optic sampling in z-cut quartz. **d**-**e**, Corresponding TKE signals of Bi_2_Se_3_ in frequency and time domain, respectively. The shaded yellow area in **d** highlights the integration region used to determine the phonon amplitude. Source data

We resonantly drive the E_u_ mode of a Bi_2_Se_3_ single-crystal epilayer (15-nm thickness), grown by molecular-beam epitaxy on Al_2_O_3_ (Methods), with circularly polarized single-cycle THz pulses with peak fields exceeding 1 MV cm^−1^ (Fig. 1c, blue shade). The normal incidence parallel to the three-fold symmetric *c* axis of Bi_2_Se_3_ defines a common axis of rotation for a consistent definition of photon and phonon helicities (Methods). We consecutively sample the transient birefringence induced by the in-plane oscillating coherent phonons with a subcycle—and thus phase-resolved—precision of a 20-fs probe pulse (Methods). This time-domain probing scheme of coherent phonons via TKE is selectively sensitive to Raman-active modes^33,34,38^. With linear probe pulse polarizations at 45° and 0° in a laboratory frame defined by the THz field components (*E*_*x*_, *E*_*y*_) measured at the sample position (Methods), we observe a strong TKE signal without signatures of transient magnetization (Supplementary Fig. 8), but dominated by a long-lived oscillatory signal (Fig. 1c). Its Fourier transform (Fig. 1d) unveils a single dominant oscillation at 4.0 THz, exactly at the resonance of the E_g_ mode^29^. Strikingly, this Raman-active phonon lies clearly outside the THz excitation spectrum (Fig. 1d, blue area) and, thus, must be nonlinearly excited either by the absorption of two photons^38^ or two coherent phonons^29–31^, as confirmed by the quadratic field dependence of the E_g_ amplitude (Extended Data Fig. 2b–e). Due to its lack of Raman activity, the E_u_ mode is not observed by TKE, but instead by linear THz absorption (Fig. 1d (pink feature) taken from Extended Data Fig. 1), in full agreement with the mutual exclusion principle in the centrosymmetric Bi_2_Se_3_ crystal.

**Extended Data Fig. 3:**
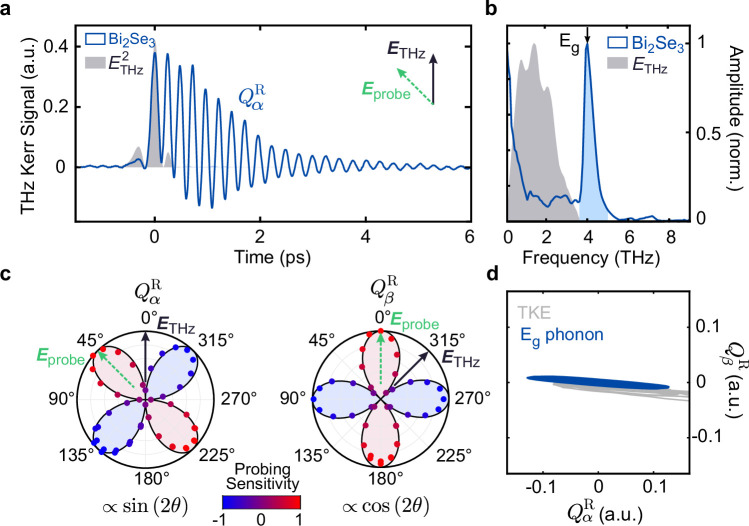
Probing phonon trajectories. **a**, Measured TKE in Bi_2_Se_3_ (blue) excited by a linearly polarized THz pulse. The probe pulse is polarized at an angle of 45^∘^ relative to the THz excitation pulse. The TKE signal around time zero includes an electronic contribution following ${{{\rm{E}}}^{2}}_{{\rm{THz}}}$ (grey shaded area). The oscillatory signal corresponds to an ionic contribution (phonon) to the TKE. **b**, Fourier transform of **a**, unveils the prominent E_g_ phonon peak at 4 THz. **c**, Spectrally-integrated (panel **b**, blue shaded area) 4 THz coherent phonon amplitude (points) as a function of probe polarization angle (green arrow) relative to vertical and 45^∘^-rotated THz excitation polarizations (black arrow), which drive orthogonal E_g_ phonon polarizations. The theoretically calculated TKE amplitude and the positive/negative sign are shown by the black line and shaded red/blue areas, respectively. **d**, Phonon trajectory of the linearly polarized E_g_ phonon measured with probe polarizations at 0^∘^ (*β*-component) and 45^∘^ (*α*-component). The grey line represents the entire TKE signal, while the blue line shows the Fourier-filtered signal of the coherent phonon. Source data

By measuring the probe polarization dependence of the phonon amplitude (Extended Data Fig. 3c), we show that the two orthogonal projections ${Q}_{\alpha }^{{\rm{R}}}$ and ${Q}_{\beta }^{{\rm{R}}}$ of the amplitude **Q**^R^ of the E_g_ mode can be separately measured by a 45° and 0° linear probe polarization, in full agreement with the two E_g_-symmetry Raman tensors^39^

1 $${R}_{\alpha }=\left(\begin{array}{cc}a & 0\\ 0 & -a\end{array}\right)\,\mathrm{and}\,\,\,\,\,\,\,\,\,\,\,{R}_{\beta }=\left(\begin{array}{cc}0 & -a\\ -a & 0\end{array}\right)$$

in the *D*_3*d*_ point group, respectively (Methods). The corresponding microscopic projections also correspond to orthogonal in-plane ion motions, associated with ‘real’ (as opposed to ‘pseudo’) microscopic angular momentum, when driven with a π/2 phase difference (Supplementary Text 1). Thus, we can trace the two-dimensional lattice trajectory of the E_g_ mode in time^26^. By implementing polarization-resolved electro-optic sampling (EOS) in α-quartz^40^, we consistently measure the full spatiotemporal trajectory of the THz electric field vector in the same plane, tracing a right-handed helicity (Fig. 1e). In the TKE signals shown in Fig. 1c, very similar oscillations are found at 45° and 0° probe polarization corresponding to the two orthogonal E_g_ components ${Q}_{\alpha }^{{\rm{R}}}$ and ${Q}_{\beta }^{{\rm{R}}}$, respectively, with identical amplitude spectra (Fig. 1d). The time-domain data (Fig. 1c, inset) and its complex Fourier transforms (Fig. 1d) unambiguously unveil a phase difference of exactly –π/2 between the orthogonal ${Q}_{\alpha }^{{\rm{R}}}$ and ${Q}_{\beta }^{{\rm{R}}}$ components. Therefore, here we demonstrate the first THz-driven preparation and simultaneous measurement of a coherent phonon angular momentum state, strictly termed an axial (colloquially coined chiral) phonon^14^. This becomes directly evident from the two-dimensional lattice trajectory shown in Fig. 1f, which strikingly reveals an opposite helicity compared with the driving field shown in Fig. 1e.

**Fig. 2:**
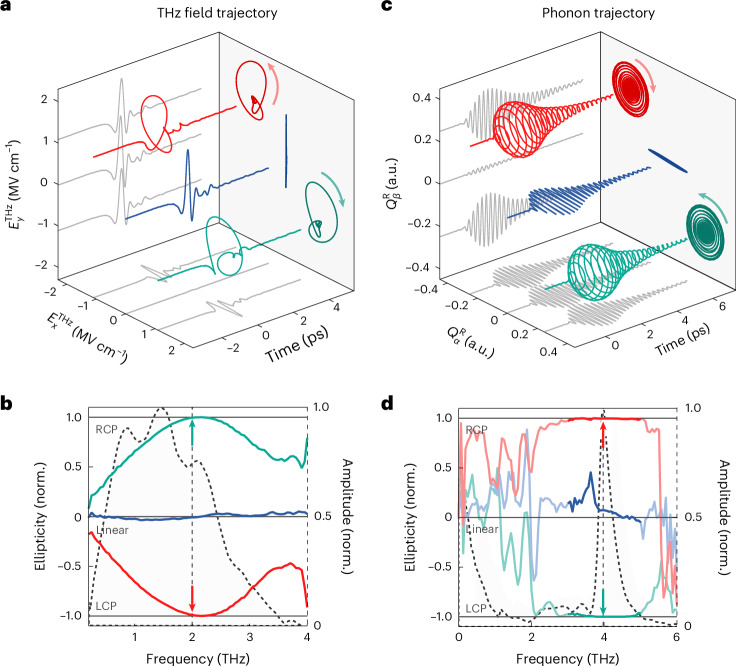
THz field and phonon trajectories and their helicity states. **a**, Polarization-resolved EOS of the THz excitation electric fields tailored by a 700-μm-thick *y*-cut quartz plate at three different azimuthal orientations. **b**, Frequency-decomposed ellipticity (coloured lines) and amplitude (dashed black line) of the excitation fields in **a**, calculated from the Stokes parameter *S*_3_ of the Fourier transform of the THz field. At around 2 THz, the THz pump pulses exhibit well-defined linear polarization (blue), RCP (green) and LCP (red). **c**, Measured E_g_ phonon trajectories for each THz polarization state from **a**. **d**, Corresponding ellipticity decompositions of the TKE signals (coloured lines) and Fourier-filtered E_g_ trajectories in **c** (darker colours). The E_g_ phonon at 4 THz shows full helicity reversal with respect to the THz electric field at 2 THz. The dashed black line represents the TKE spectral amplitude. Source data

For a systematic investigation of the phonon angular momentum transfer, we turn to tailored helical THz driving fields. By transformation of the exact THz field trajectories (Fig. 2a) from time into frequency domain, and from a linear (*E*_*x*_, *E*_*y*_) basis into a circular $({E}_{\left|{\rm{R}}\right\rangle },{E}_{\left|{\rm{L}}\right\rangle })$ basis (Fig. 2b), we follow the complex evolution of the helicity state of a single cycle across its broad spectrum. With this information, we customized a *y*-cut quartz waveplate plate (thickness, 700 μm) to provide a pure right-handed circularly polarized (RCP) or left-handed circularly polarized (LCP) THz helicity state at 2 THz (Fig. 2b). The three distinct fields with net helicity *l*_s_ = −1, 0, 1 at 2 THz (Fig. 2a) are then used to prepare the IR-active E_u_ phonon in a prescribed coherent angular momentum state.

By comparing the resulting E_g_ phonon trajectories (Fig. 2c), we find perfectly opposite phonon helicities compared with the respective THz driving fields (Fig. 2a). Transforming the phonon trajectories into the frequency domain and circular basis, the Stokes parameter of the E_g_ phonons, ${S}_{3}(\Omega )=(| {Q}_{\left|R\right\rangle }^{{\rm{R}}}(\Omega ){| }^{2}-| {Q}_{\left|L\right\rangle }^{{\rm{R}}}(\Omega ){| }^{2})/| {Q}^{{\rm{R}}}(\Omega ){| }^{2}$, unveils pure helicities of two opposite angular momentum states across the full phonon bandwidth. Accordingly, we find a helicity switch: for the excitation with an RCP (LCP) field at 2 THz, the Raman-active phonon at 4 THz possesses perfect LCP (RCP) and, thus, opposite helicity (Fig. 2a,c, arrows). In the following, we will address two arising questions. What is the nonlinear excitation mechanism of the observed phonon angular momentum state? How is crystal angular momentum conserved, leading to the switched helicity?

## Pathways for angular momentum transfer

**Fig. 3:**
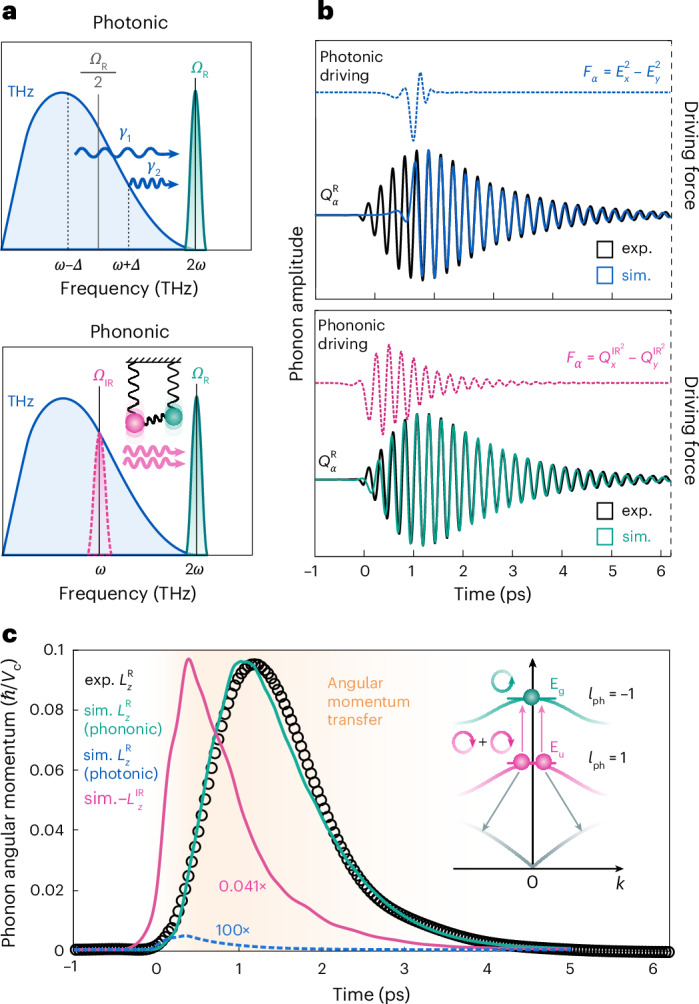
Angular momentum transfer channels and dynamics. **a**, Schematic of the nonlinear excitation pathways. ‘Photonic’ THz sum-frequency excitation (top) involves two spectral THz components driving a Raman-active phonon at their sum frequency. ‘Phononic’ sum-frequency ionic Raman scattering (bottom) involves a resonantly driven IR-active phonon (E_u_) coupling nonlinearly to a Raman-active mode (E_g_). **b**, Modelled driving forces *F*_*α*_ (dotted lines) and the resulting phonon amplitudes ${Q}_{\alpha }^{{\rm{R}}}$ (blue, green) for photonic (top; offset in time to match the phonon decay dynamics) and phononic (bottom) pathways compared with experimental data (black) under RCP THz excitation. **c**, Quantitative ab initio modelling of angular momentum dynamics for all involved modes and pathways. Experimentally measured angular momentum dynamics ${L}_{z}^{{\rm{R}}}$ (black circles) are normalized to the peak magnitude of the calculated phononic pathway ${L}_{z}^{{\rm{R}}}$ (green). The shaded orange region highlights angular momentum transfer from E_u_ (pink) to E_g_, resulting in a delayed rise time and three-orders-of-magnitude higher angular momentum transfer (green) compared with the photonic pathway (blue). Inset: 3% of the E_u_ angular momentum is transferred to the E_g_ mode, and the remaining probably dissipates into lower-energy and higher-momentum modes (grey). exp., experimental; sim., simulated. Source data

Generally, the purely Raman-active E_g_ mode can be driven by nonlinear photonic or nonlinear phononic pathways^30,33,38^ (Fig. 3a). In the photonic case, pairs of photons within the broad THz excitation spectrum excite the E_g_ mode via their sum-frequency polarization^38^. For the nonlinear phononic coupling, the initially driven IR-active E_u_ phonon **Q**^IR^ couples via the ${({Q}^{{\rm{IR}}})}^{2}{Q}^{{\rm{R}}}$ anharmonic term in the lattice potential *V*(**Q**^IR^, **Q**^R^) to the Raman-active mode, known as sum-frequency ionic Raman scattering^30^. In the photonic case, the driving force for the E_g_ mode is given by **E** ⋅ *R*_*α*_**E** and **E** ⋅ *R*_*β*_**E** and is, thus, proportional to ${E}_{x}^{2}-{E}_{y}^{2}$ and −2*E*_*x*_*E*_*y*_ for the ${Q}_{\alpha }^{{\rm{R}}}$ and ${Q}_{\beta }^{{\rm{R}}}$ components, respectively. In the *D*_3*d*_ point group, to which Bi_2_Se_3_ belongs, the lowest-order anharmonic lattice potential that couples the modes of interest is $V({{\bf{Q}}}^{{\rm{IR}}},{{\bf{Q}}}^{{\rm{R}}})=c[{({Q}_{y}^{{\rm{IR}}})}^{2}-{({Q}_{x}^{{\rm{IR}}})}^{2}]{Q}_{\alpha }^{{\rm{R}}}+2c\,{Q}_{x}^{{\rm{IR}}}{Q}_{y}^{{\rm{IR}}}{Q}_{\beta }^{{\rm{R}}}+O({Q}^{4})$, where *c* is the anharmonic coupling constant determined using our density functional theory (DFT) calculations (Methods and Supplementary Texts 2 and 3). Its derivative with respect to the Raman-active phonon coordinate −∂*V*/∂**Q**^R^ yields the ionic driving forces ${F}_{\alpha }=c[{({Q}_{x}^{{\rm{IR}}})}^{2}-{({Q}_{y}^{{\rm{IR}}})}^{2}]$ and ${F}_{\beta }=-2c\,{Q}_{x}^{{\rm{IR}}}{Q}_{y}^{{\rm{IR}}}$, respectively. Therefore, both driving mechanisms obey the same symmetry dictated by the *D*_3*d*_ point group. One way to distinguish these two pathways is by their respective driving force dynamics, leading to different phonon dynamics when solving the respective **Q**^R^ equations of motion, as shown for ${Q}_{\alpha }^{{\rm{R}}}$ in Fig. 3b. We find that the rise of the experimental trace, matching the E_u_ lifetime as determined from THz transmission (Extended Data Fig. 1), is only reproduced by the phononic mechanism, pinpointing to the excited IR-active E_u_ phonon as the primary driving force of the Raman-active phonon.

To quantify the contribution of nonlinear phonon–phonon angular momentum transfer, we calculate the microscopic angular momentum ${L}_{z}^{{\rm{R}}}$ of the excited E_g_ phonon per unit cell from the photonic versus the phononic driving pathway. We compute all involved light–matter coupling parameters, Raman tensors and anharmonic coupling parameters up to the fourth order using DFT (Methods). These DFT calculations and complementary symmetry considerations (Supplementary Text 1) attribute ‘real’ angular momentum to both modes, independent of the azimuthal sample orientation and resulting from the real-space atomic motions in our laboratory frame defined by the vectorial THz field^41^ (Supplementary Video 3). Strikingly, our ab initio model confirms a three-orders-of-magnitude larger angular momentum transfer from the E_u_ mode to the E_g_ mode compared with the direct driving of the E_g_ mode via the photonic excitation process (Fig. 3c). Taking into account the experimentally determined phonon coherence times of 1.6 ps and 1.1 ps for the IR- and Raman-active mode, respectively, and the measured THz field trajectory (Fig. 1e) leads to a remarkable agreement with the measured phonon angular momentum dynamics ${{\bf{L}}}^{{\rm{R}}}(t)={{\bf{Q}}}^{{\rm{R}}}(t)\times {\dot{{\bf{Q}}}}^{{\rm{R}}}(t)$ (Fig. 3c). We find that ~3% of the absolute E_u_ angular momentum is upconverted to the E_g_ mode at the Γ point, which strongly depends on the anharmonic coupling coefficient between the E_g_ and E_u_ modes, their frequency-matching conditions and the angular momentum loss to the acoustic modes taken into account by the phenomenological damping constants. Accordingly, the observed coherent process inverts the usual equilibration flow of angular momentum and provides the first direct observation of anharmonic phonon–phonon angular momentum transfer. The remaining E_u_ angular momentum probably dissipates into lower-energy and higher-momentum acoustic modes (Fig. 3c, inset), which is generally permitted by an equivalent four-phonon scattering term ${({Q}^{{\rm{IR}}})}^{2}Q(-k)Q(k)$ of the anharmonic lattice potential^35^. In the following, we will discuss the underlying angular momentum conservation rules of the observed three-phonon scattering in the classical field and quantum particle picture.

## Conservation of crystal angular momentum

**Fig. 4:**
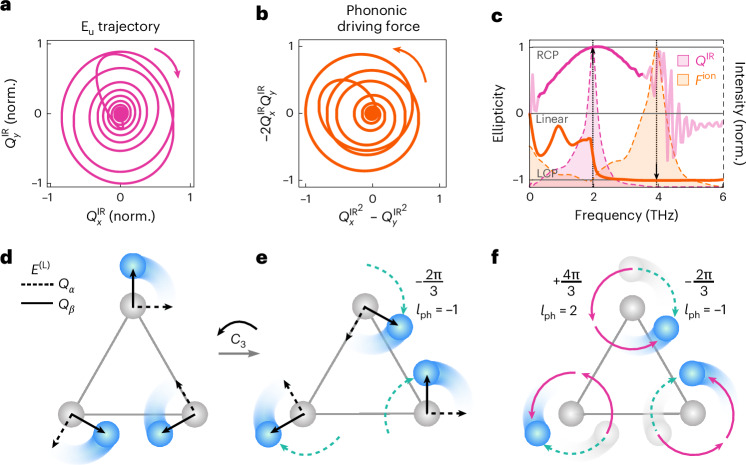
Conservation of phonon angular momentum. **a**–**c**, Classical field picture: helicity reversal dictated by the symmetry of anharmonic lattice potential. **a**, E_u_ phonon trajectory calculated from the experimental RCP THz excitation field. **b**, Corresponding phononic driving force for the E_g_ mode, rotating in the opposite direction. **c**, Frequency-decomposed ellipticity of the E_u_ phonon *Q*^IR^ (pink) and its phononic driving force *F*^ion^ (orange) showing opposite helicities at 2 and 4 THz, respectively. **d**–**f**, PAM conservation in the particle picture under three-fold rotational invariance. **d**, Solid and dashed black arrows indicate the orthogonal components of a doubly degenerate E mode in a triangular lattice. All lattice sites are in phase in the case of a Γ mode. **e**, Under three-fold rotation, the crystal remains invariant up to a phase factor of 2π*l*_ph_/3, which defines the PAM to *l*_ph_ = −1 here. **f**, Equivalence of *l*_ph_ = −1 and *l*_ph_ = +2 in three-fold symmetric groups. *l*_ph_ = +2 is provided by the two-phonon absorption, corresponding to rotational three-phonon Umklapp scattering. Source data

In the classical picture, the discrete rotational invariance of the crystal is captured by the point-group symmetry and imprinted in the anharmonic lattice potential *V*(**Q**^IR^, **Q**^R^). The lowest-order symmetry-allowed squared-linear ${({Q}^{{\rm{IR}}})}^{2}{Q}^{{\rm{R}}}$ coupling term, thus, dictates opposite helicities of the E_u_ mode and its nonlinear driving force, as modelled in Fig. 4a,b, respectively. This becomes more apparent through transformation to a circular basis ${Q}_{\left|{\rm{R}}\right\rangle /\left|{\rm{L}}\right\rangle }^{{\rm{IR}}}=({Q}_{x}^{{\rm{IR}}}\pm {\rm{i}}{Q}_{y}^{{\rm{IR}}})/\sqrt{2}$, which turns the driving force into ${F}_{\left|{\rm{R}}\right\rangle /\left|{\rm{L}}\right\rangle }=({F}_{\alpha }\pm {\rm{i}}{F}_{\beta })/\sqrt{2}$ and thus

2 $${F}_{\left|{\rm{R}}\right\rangle /\left|{\rm{L}}\right\rangle }=c\,{\left({Q}_{\left|{\rm{L}}\right\rangle /\left|{\rm{R}}\right\rangle }^{\mathrm{IR}}\right)}^{2}.$$

From this, it is clear that the nonlinear phonon–phonon coupling doubles the E_u_ frequency and inverts the helicity to enforce energy and angular momentum conservation, respectively (Fig. 4c).

Turning to the quasiparticle picture, we evoke the definition of phonon crystal (or pseudoangular momentum (PAM) *l*_ph_) as a conserved quantity under the *C*_3_ rotational symmetry of Bi_2_Se_3_ (ref. ^42^)

3 $$\widehat{{C}_{3}}{\phi }_{l}(\varphi )={{\rm{e}}}^{{\rm{i}}\frac{2\pi }{3}{l}_{\mathrm{ph}}}{\phi }_{l}(\varphi ),$$

where *ϕ*_*l*_ is the wave function of the phonon at the high-symmetry point *k* = 0. It is sufficient to depict the two orthogonal components of the E modes under *C*_3_ symmetry on the simplest triangle lattice^26^ (Fig. 4d). As the axial mode is a superposition of two linearly polarized Γ-point modes, the *Q*_*α*_ and *Q*_*β*_ components have identical phase relations at each of the three lattice sites. After performing an in-plane *C*_3_ rotation (Fig. 4e), an additional 2π/3 phase factor has to be applied to obtain the initial eigenstate of Fig. 4d. In other words, the ions have to go a third of their oscillation period backwards in time to reach the same position as a *C*_3_ rotation of a time snapshot. By definition in equation (3), the PAM is *l*_ph_ = −1 per phonon for the observed LCP E mode. According to the same equation, in this *C*_3_ symmetry, *l*_ph_ = −1 is equivalent to *l*_ph_ = 2, corresponding to an Umklapp process in a rotational Brillouin zone^36,37,42^. Or simply speaking, as depicted in Fig. 4f, we may either move 4π/3 of the phonon phase forward or 2π/3 backwards in time to reconstruct the phonon wave function after the *C*_3_ operation. In nonlinear phononics, the E_g_ amplitude scales with the square of the E_u_ driving mode and, thus, inherits twice its phase: ${Q}^{{\rm{R}}}\propto {({Q}^{{\rm{IR}}})}^{2}={{\rm{e}}}^{{\rm{i}}2{\phi }_{{\rm{IR}}}}{({\widehat{Q}}^{{\rm{IR}}})}^{2}$. In the analogue particle picture, energy and PAM conservation of two annihilated E_u_ phonon quanta with ${l}_{{\rm{ph}}}^{{\rm{IR}}}=1$ dictate that the E_g_ phonon must carry twice the energy and PAM of the E_u_ phonon, and thus, ${l}_{{\rm{ph}}}^{{\rm{R}}}=2{l}_{{\rm{ph}}}^{{\rm{IR}}}=-1$. The experimentally observed exact reversal of phonon helicity would violate angular momentum conservation in free space (continuous rotational invariance), but perfectly obeys the crystal (or pseudo) angular momentum conservation under discrete *C*_3_ rotational invariance in the investigated *a*–*b* plane of Bi_2_Se_3_. This phenomenon can be considered as rotational phonon–phonon Umklapp scattering, being the nonlinear phononics analogue of the rotational Umklapp process in nonlinear optics^36^ and the angular momentum counterpart of linear momentum phonon–phonon Umklapp scattering governing solid-state physics^1^.

## General implications

Most generally, our experimental observation confirms the long-standing hypothesis: phonon–phonon angular momentum transfer is permitted via lattice anharmonicity and conserves crystal angular momentum. This has far-reaching implications for spin relaxation phenomena, such as ultrafast demagnetization, and eventually identifies one of the unknown intermediate steps of the (ultrafast) Einstein–de Haas effect^2,3^. Our findings suggest that ‘natural’ incoherent angular momentum dissipation from higher-energy to lower-energy (and potentially higher linear momentum) modes follows anharmonic coupling mechanisms^43^ equivalent to the anharmonic upconversion demonstrated in this work. This motivates higher-momentum probing techniques like ultrafast electron^44,45^ or X-ray^43^ diffraction to study the incoherent relaxation of phonon–phonon angular momentum transfer.

The demonstrated coherent angular momentum and energy upconversion, moreover, opens the field of axial nonlinear phononics for the selective control of chiral states of matter. Our observations in the topological insulator Bi_2_Se_3_ directly enable studies on phonon-angular-momentum-driven control of topological surface states via spin-selective electron–phonon scattering^46^ and will stimulate novel investigations of phonon-driven topology switching^47^. Furthermore, the witnessed rotational phonon–phonon Umklapp scattering may play a similarly fundamental role for angular momentum dissipation and thermal Hall conductivity^15^, as the linear momentum Umklapp processes does in heat transport^1^. Our observation of back-folded PAM states motivates further studies on phonon rotational Bloch functions and phonon-driven Floquet matter^48,49^, with potential implications on topological phonon states^50^, active control of spin-valley polarization^13^ or phonon orbital angular momentum states^51^.

In conclusion, our results experimentally confirm the full analogy to energy and linear momentum relaxation in solids. The anharmonicity of the crystal lattice mediates the transfer and conservation of phonon angular momentum. In the classical picture, the discrete rotational symmetry imprinted in the anharmonic lattice potential enforces the observed helicity switch between the two coupled modes. In the quasiparticle picture, crystal angular momentum is transferred and conserved by a rotational Umklapp process during three-phonon scattering. This concept can be extended to any higher-order lattice anharmonicity and multiphonon scattering abundant in nature and, therefore, completes the solid-state physics picture of axial and chiral phonons as quasiparticles responsible for carrying and conserving crystal angular momentum. In the future, the axial nonlinear phononics established here will provide a precise handle for ultrafast control over spins, topologies and chiral quasiparticles.

## Methods

### Sample details

The sample was grown on a sapphire(0001) substrate by means of molecular-beam epitaxy. Before the growth of the Bi_2_Se_3_ epilayer, the sapphire substrate was annealed at 900 °C for 30 min. Subsequently, the substrate temperature was reduced to 270 °C for the growth of the Bi_2_Se_3_ film, which took place in a Se overpressure regime. The Bi and Se partial pressures amount to 5.3 × 10^−8^ mbar and 5.8 × 10^−7 ^mbar, respectively, which results in a growth rate of 18.3 nm h^−1^.

The sample was ex situ characterized by X-ray diffraction (XRD), using a Rigaku SmartLab XRD system. The film thickness was determined by means of X-ray reflectivity (Supplementary Fig. 5). Numerous oscillations are seen in the X-ray reflectivity curve, evidencing the high surface and interface quality of the Bi_2_Se_3_ film. From the periodicity of the oscillations, a Bi_2_Se_3_ film thickness of 15.0 nm is determined. To obtain information about the crystal structure of the epilayer, XRD measurements were performed (Supplementary Fig. 6). Besides the three peaks stemming from the sapphire substrate, a total of seven (0,0,*l*) peaks from the Bi_2_Se_3_ epilayer are seen, proving the single-crystal nature of the Bi_2_Se_3_ film with the *c* axis in the growth direction.

### Experimental setup

The experimental setup is shown in Extended Data Fig. 2a. Intense single-cycle THz pulses (1.5-THz central frequency, 2.0-THz spectral full-width at half-maximum, 1-kHz repetition rate) with peak fields exceeding 1.5 MV cm^−1^ are generated by optical rectification in LiNbO_3_ using the tilted pulse front technique^52^. Synchronized optical probe pulses (790-nm central wavelength, 1.6-nJ pulse energy, 80-MHz repetition rate, 20-fs pulse duration) are collinearly incident on the sample at normal incidence. The linear polarization of the probe is controlled using a half-wave plate to resolve two orthogonal components of the E_g_ phonon mode (Fig. 1f). The intense THz pulses induce a transient birefringence in the sample, which alters the polarization of the transmitted optical probe pulses (TKE^32^). Coherently excited Raman-active phonons also contribute to this transient birefringence by modulating linear susceptibility^34,53^. A balanced detection scheme comprising a half-wave plate, Wollaston prism and balanced photodiodes is used to measure this change in polarization.

### Helicity-tailored THz fields

**Extended Data Fig. 4:**
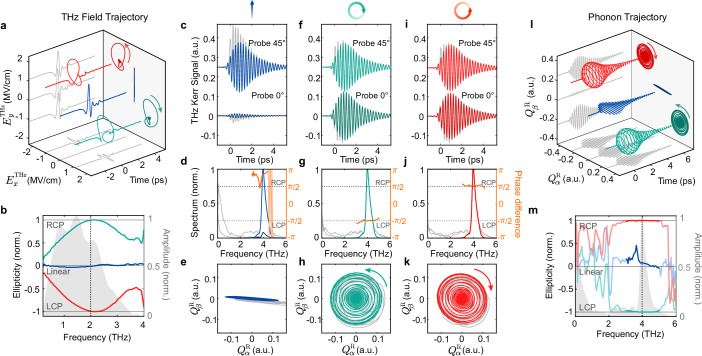
Lattice trajectory control by circular THz excitation in Bi_2_Se_3_. **a**, Polarization-resolved EOS of the incident THz electric field transmitted through 700 *μ*m-thick y-cut quartz at three azimuthal crystal orientations. **b**, Frequency-resolved helicity states of the THz fields in **a**. The shaded grey area represents the THz spectral amplitude. **c**-**k**, Measured E_g_ trajectories under linear (blue), right-circular (green) and left-circular (red) THz excitation in Bi_2_Se_3_. **c**, Full TKE signals (grey) and Fourier-filtered phonon contributions (blue) measured with probe polarized at 0^∘^ (${S}_{{0}^{\circ }}$) and 45^∘^ (${S}_{4{5}^{\circ }}$), vertically offset for clarity, corresponding to two orthogonal E_g_ phonon components. **d**, Spectral amplitudes of signals in **c**, with the relative phase difference of the TKE signals ${S}_{4{5}^{\circ }}-{S}_{{0}^{\circ }}$ (orange). **e**, Corresponding vectorial phonon trajectory. **f**-**k**, Same data set as **c**-**e**, but for right- and left-circular THz excitation. **l**, Coherent phonon trajectories for each THz helicity in **a**, extracted by Fourier-filtering the phonon contribution in the TKE signal. **m**, Frequency-resolved helicity of the E_g_ phonon from **l**. The grey background represents the TKE spectrum, with the peak at 4 THz corresponding to the Raman-active E_g_ phonon. Source data

**Extended Data Fig. 5:**
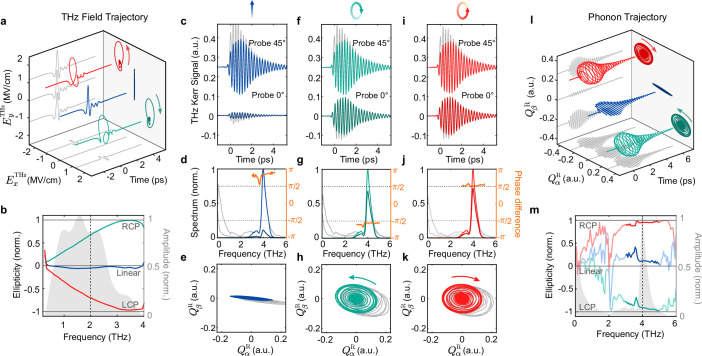
Lattice trajectory control by arbitrary elliptical THz excitation in Bi_2_Se_3_. Same as Extended Data Fig. 4, but for the incident THz electric field transmitted through 380 *μ*m-thick y-cut quartz waveplate. **a**, Polarization-resolved EOS of the incident THz electric fields. **b**, Frequency-resolved helicity states of the THz fields in **a**. The shaded grey area represents the THz spectral amplitude. **c**-**k**, Measured E_g_ phonon trajectories under linear (blue), right-handed elliptical (green) and left-handed elliptical (red) THz excitation in Bi_2_Se_3_. **l**, Coherent E_g_ phonon trajectories for each THz helicity in **a**, extracted by Fourier-filtering the phonon contribution in the TKE signal. **m**, Frequency-resolved helicty of the E_g_ phonon from **l**. The grey background represents the TKE spectrum, with the peak at 4 THz corresponding to the Raman-active E_g_ phonon. Source data

To control the THz polarization state, we use a birefringent 700-μm-thick *y*-cut quartz waveplate that imparts a frequency-dependent relative phase retardation between the orthogonal fast and slow crystalline axes (Supplementary Fig. 7a,b). Due to the highly polychromatic nature of a single-cycle THz pulse, the waveplate generates a continuum of elliptical polarization states across the broad spectrum constituting the pulse, but is tailored to have a particular thickness to generate a desired circular polarization state at 2 THz, where the E_u_ phonon resonance is situated. By orienting the quartz waveplate to 0°, –45° and +45° with respect to the vertical incident THz polarization, the transmitted THz polarization state is toggled between linear polarization, RCP and LCP at 2 THz (Extended Data Fig. 4a,b). In addition, to investigate arbitrary lattice trajectories (Extended Data Fig. 5l), we use a 380-μm-thick *y*-cut quartz waveplate (Supplementary Fig. 7c,d) generating an elliptically polarized THz beam (Extended Data Fig. 5a,b).

### Measuring E_u_ phonon lifetime via THz transmission

We perform THz time-domain spectroscopy (Extended Data Fig. 1a) to measure the THz transmittance of a Bi_2_Se_3_ film. A spintronic THz emitter (TeraSpinTech T-Spin2) generates an ultrabroadband THz pulse^54^, which is focused onto the sample in the first focal position. The transmitted THz pulse is then refocused onto a 30-μm-thick GaP crystal for EOS. To determine the intrinsic THz transmittance ${T}_{{{\rm{Bi}}}_{2}{{\rm{Se}}}_{3}}(\omega )$ of Bi_2_Se_3_, we conduct two measurements: (1) the THz transmission of a 0.3-μm-thick Bi_2_Se_3_ film grown on a 500-μm sapphire substrate and (2) a reference THz transmission through an identical 500-μm-thick sapphire wafer (Extended Data Fig. 1b,c). Taking the ratio of these spectra eliminates the detector response function of the GaP crystal and partially removes the substrate contribution and, therefore, contains the intrinsic THz transmittance properties of Bi_2_Se_3_ (Extended Data Fig. 1d). The resulting characteristic absorption feature at 2 THz confirms the dominant absorption due to the resonant excitation of the IR-active E_u_ phonon mode^28^.

We identify an analytical expression for the THz transmission function ${T}_{{{\rm{Bi}}}_{2}{{\rm{Se}}}_{3}}(\omega )$ based on Fresnel coefficients, incorporating the Bi_2_Se_3_ dielectric function to extract the E_u_ phonon frequency *ω*_IR_ and damping rate *γ*_IR_, which defines the phonon lifetime *τ*_IR_ = 1/(2π*γ*_IR_). The derived transmission function ${T}_{{{\rm{Bi}}}_{2}{{\rm{Se}}}_{3}}(\omega )$ (Supplementary Text 4 provides the derivation) accounts for multiple internal reflections within Bi_2_Se_3_, closely matching the measured Bi_2_Se_3_ transmittance (Extended Data Fig. 1d). By modelling the experimental data, we determine the E_u_ phonon parameters to be *ω*_IR _= 1.97 THz and *γ*_IR _= 0.2 THz, corresponding to the phonon lifetime *τ*_IR _= 0.79 ps.

### Helicity-state characterization

In our experimental geometry, the co-propagating pump and probe pulses arrive at normal incidence on the Bi_2_Se_3_ surface, establishing a unique optical axis that coincides with the crystallographic *c* axis of the hexagonal unit cell (confimed by XRD; Supplementary Fig. 6) and, thus, with its *C*_3_-symmetry axis. The transverse THz electric field components, *E*_*x*_(*t*) and *E*_*y*_(*t*), measured with full sign sensitivity at the sample position^40^, define the laboratory reference frame and its handedness. This configuration ensures a single, well-defined rotation axis for all polarization analyses, allowing the helicity of both THz field and coherently driven E_g_ phonon to be characterized and compared within the same coordinate system.

We characterize the complex helicity states of the single-cycle THz pulses (Fig. 2b) and arbitrarily polarized phonon states (Fig. 2d) by computing the ellipticity parameter in the frequency domain. The ellipticity, which quantifies the relative contribution of circular polarization components, is calculated as the normalized third Stokes parameter *S*_3_(*ω*), independently evaluated at each frequency. To achieve this, the electric field components *E*_*x*_(*ω*) and *E*_*y*_(*ω*) in the Cartesian basis $(\widehat{x},\widehat{y})$ are transformed into the circular basis $(\widehat{R},\widehat{L})$. The circular basis is defined according to the standard optics convention for RCP and LCP, and the sign of the phonon PAM (equation (3)) is chosen accordingly. The corresponding field components in the circular basis are computed as ${E}_{{\rm{R}}}(\omega )=({E}_{x}(\omega )+{\rm{i}}{E}_{y}(\omega ))/\sqrt{2}$ and ${E}_{{\rm{L}}}(\omega )=({E}_{x}(\omega )-{\rm{i}}{E}_{y}(\omega ))/\sqrt{2}$. The ellipticity parameter *S*_3_(*ω*) is then determined as *S*_3_(*ω*) = (∣*E*_R_(*ω*)∣^2^ − ∣*E*_L_(*ω*)∣^2^)/∣*E*(*ω*)∣^ 2^, where ∣*E*(*ω*)∣^ 2^ = ∣*E*_R_(*ω*)∣^2^ + ∣*E*_L_(*ω*)∣^2^. The parameter takes values in the range [−1, 1], with *S*_3_(*ω*) = 1 corresponding to pure RCP, *S*_3_(*ω*) = −1 to pure LCP and *S*_3_(*ω*) = 0 to any possible linear polarization. The same procedure is applied to characterize the polarization state of a phonon with the amplitude ${{\bf{Q}}}^{{\rm{R}}}(\omega )=({Q}_{\alpha }^{{\rm{R}}}(\omega ),{Q}_{\beta }^{{\rm{R}}}(\omega ))$. This approach provides a frequency-resolved description of the helicity states for both THz pulses and phonon modes.

### Probing E_g_ phonon trajectories

The coherent phonon dynamics of the E_g_ mode are monitored via the TKE, where the intense THz pulses induce transient optical birefringence in Bi_2_Se_3_ sample, mediated by the coherent lattice vibration. As the optical probe pulse propagates through the sample, its polarization undergoes a rotation proportional to the instantaneous amplitude of the coherent phonon. We note that the temporal evolution of the E_g_ mode is, thus, measured by varying the time delay between the THz pump and optical probe pulses.

Importantly, the TKE originating from coherent phonons involves a Raman-type probing mechanism^53^, in which the THz pump and optical probe fields couple via the third-order nonlinear susceptibility, analogous to stimulated Raman scattering experiments. In this regard, the Raman tensor formalism provides a framework for understanding the probing of phonon trajectories.

The probing signal *S*^pr^ of a particular phonon’s amplitude is dependent on the in-plane azimuthal angle of the probe pulse polarization, and the signal amplitude is determined by the phonon symmetry encoded in the Raman tensor. This Raman-type probing via transient birefringence can be expressed as ${S}^{{\rm{pr}}}(\theta )\propto {\widehat{e}}_{i}^{\perp {\rm{pr}}}(\theta )Q(t){R}_{ij}^{Q}{\widehat{e}}_{j}^{{\rm{pr}}}(\theta )$, where ${\widehat{e}}^{{\rm{pr}}}(\theta )$ and ${\widehat{e}}^{\perp {\rm{pr}}}(\theta )$ are the polarization vector of the incident probe pulse oriented at the angle *θ* in the laboratory frame and a unit vector perpendicular to it, respectively. ${R}_{ij}^{Q}$ is the Raman tensor of a given phonon mode *Q*(*t*).

In the case of a doubly degenerate mode, such as the E_g_ mode, two distinct Raman tensors are involved in the probing process, corresponding to the two orthogonal components that form a basis for polarization-resolved detection. In this representation, the probing signal is given by ${S}^{{\rm{pr}}}\propto {\widehat{e}}_{i}^{\perp {\rm{pr}}}({Q}_{\alpha }(t){R}_{ij}^{{Q}_{\alpha }}+{Q}_{\beta }(t){R}_{ij}^{{Q}_{\beta }}){\widehat{e}}_{j}^{{\rm{pr}}}(\theta )$, where *Q*_*α*_(*t*) and *Q*_*β*_(*t*) are the classical amplitudes of the orthogonal phonon components, ${R}_{ij}^{{Q}_{\alpha }}$ and ${R}_{ij}^{{Q}_{\beta }}$ are the corresponding Raman tensors in the laboratory frame $(\widehat{x},\widehat{y})$. For the E_g_ phonon mode, the Raman tensors of two orthogonal projections *Q*_*α*_(*t*) and *Q*_*β*_(*t*) are given by^39,55^

4 $${R}_{\alpha }=\left(\begin{array}{cc}a & 0\\ 0 & -a\end{array}\right),\,\,\,\,\,\,\,\,\,{R}_{\beta }=\left(\begin{array}{cc}0 & -a\\ -a & 0\end{array}\right).$$

Therefore, for a probe pulse with an incident polarization $${\widehat{e}}^{\mathrm{pr}}=$$$$(-\sin \theta ,\cos \theta )$$, which is aligned vertically at *θ* = 0°, the resulting probing signal is given by $${S}^{{\rm{pr}}}(t,\theta )\propto {Q}_{\alpha }(t)\sin 2\theta +{Q}_{\beta }(t)\cos 2\theta$$. This angular dependence reflects the quadrupolar symmetry of the E_g_ phonon mode, and enables the selective detection of its orthogonal components. By aligning the probe polarization to *θ* = 45° and *θ* = 0°, the contribution of *Q*_*α*_(*t*) and *Q*_*β*_(*t*) can be isolated (Extended Data Fig. 3c), respectively, allowing for the reconstruction of the E_g_ phonon trajectory^26^.

Note that all TKE and polarization-resolved EOS measurements use the same probe polarizations to resolve the *x* and *y* components of the E_g_ phonon state and THz pump field. This results from the shared three-fold rotational-symmetry property of Bi_2_Se_3_ (*D*_3*d*_ point group) and the α-quartz EOS detector crystal (D_3_ point group). The EOS signal ${S}^{{\rm{EOS}}}(t,\theta )\propto {E}_{x}^{{\rm{THz}}}(t)\sin 2\theta +{E}_{y}^{{\rm{THz}}}(t)\cos 2\theta$ follows directly from the second-order nonlinear polarization **P**^(2)^ defined by the *χ*^(2)^ tensor of D_3_ symmetry^40^. *S*^EOS^ and *S*^pr^ exhibit the same angular dependence, ensuring that the polarizations of both E_g_ phonon and THz field are measured consistently in the same reference frame, enabling the unambiguous identification of helicity reversal.

### Excitation pathways of the E_g_ mode

The higher-frequency Raman-active E_g_ phonon mode can be driven via two distinct pathways: photonic sum-frequency excitation^38^ and sum-frequency ionic Raman scattering^30^ through anharmonic coupling with IR-active E_u_ phonon. The temporal evolution of the doubly degenerate E_g_ phonon amplitude can be described classically by a system of two equations of motion with the vectorial driving force **F**(*t*):

5 $${\ddot{Q}}_{i}^{{\rm{R}}}+{\gamma }_{{\rm{R}}}{\dot{Q}}_{i}^{{\rm{R}}}+{\omega }_{{\rm{R}}}^{2}{Q}_{i}^{{\rm{R}}}={F}_{i}(t),\,i=\{\alpha ,\beta \},$$

where **Q**^R^ is the coherent amplitude of the E_g_ mode.

In the photonic pathway, the THz field **E**^THz^ = (*E*_x_, *E*_y_) directly couples to the Raman tensors of the E_g_ mode, yielding a driving force

6 $${{\bf{F}}}_{{\rm{R}}}^{{\rm{ph}}}\propto \left(\begin{array}{c}{R}_{\alpha }^{ij}{E}_{i}{E}_{j}\\ {R}_{\beta }^{ij}{E}_{i}{E}_{j}\end{array}\right)=a\left(\begin{array}{c}{E}_{x}^{2}-{E}_{y}^{2}\\ -2{E}_{x}{E}_{y}\end{array}\right).$$

In the phononic pathway, the driving force originates from the anharmonic coupling between the Raman-active E_g_ mode and the IR-active E_u_ mode at half its frequency. The lowest-order, symmetry-allowed anharmonic potential coupling the Raman-active E_g_ and the IR-active E_u_ modes is given by

7 $$V({{\bf{Q}}}^{{\rm{IR}}},{{\bf{Q}}}^{{\rm{R}}})=c[{({Q}_{y}^{{\rm{IR}}})}^{2}-{({Q}_{x}^{{\rm{IR}}})}^{2}]{Q}_{\alpha }^{{\rm{R}}}+2c{Q}_{x}^{{\rm{IR}}}{Q}_{y}^{{\rm{IR}}}{Q}_{\beta }^{{\rm{R}}}+O({Q}^{4}),$$

where **Q**^IR^ and **Q**^R^ are coherent phonon amplitudes of the E_u_ and E_g_ phonon states, respectively. This simplest form of the potential results in a phononic driving force on the E_g_ mode

8 $${{\bf{F}}}_{{\rm{R}}}^{{\rm{ion}}}\propto \left(\begin{array}{c}-\partial V/\partial {Q}_{\alpha }^{{\rm{R}}}\\ -\partial V/\partial {Q}_{\beta }^{{\rm{R}}}\end{array}\right)=c\left(\begin{array}{c}{({Q}_{x}^{{\rm{IR}}})}^{2}-{({Q}_{y}^{{\rm{IR}}})}^{2}\\ -2{Q}_{x}^{{\rm{IR}}}{Q}_{y}^{{\rm{IR}}}\end{array}\right).$$

Spectrally, the phononic driving force ${{\bf{F}}}_{{\rm{R}}}^{{\rm{ion}}}(\omega )$ is given by the convolution of two Lorentzians *Q*^IR^(*ω*) centred each at *Ω*_IR_, yielding a spectrum centred at 2*Ω*_IR_ with linewidth 2*γ*_IR_ (Fig. 4c). The Raman-active mode is efficiently driven via the anharmonic ${Q}_{{\rm{IR}}}^{2}{Q}_{{\rm{R}}}$ coupling when its frequency *Ω*_R_ overlaps with the spectral window of the driving force, reaching the maximum coupling at *Ω*_R_ = 2*Ω*_IR_. If this frequency window is not matched, higher-order anharmonic terms may still permit the coupling.

The photonic (equation (6)) and phononic (equation (8)) driving forces exhibit identical spatiorotational symmetry, as the THz field and the IR-active E_u_ mode both transform as a vector representation within the *D*_3*d*_ point group of the Bi_2_Se_3_ crystal lattice. Although the spatial symmetries of the forces are the same, their temporal profiles differ: the photonic driving force follows the duration of the single-cycle THz pulse, whereas the phononic driving force is governed, for short THz excitation pulses, by the lifetime of the IR-active E_u_ phonon, setting the timescale for phonon–phonon coupling (Fig. 3b).

The different durations of the driving forces result in distinctive rise times in the E_g_ phonon evolution. To identify the dominant excitation mechanism, we analyse the rise time of the measured E_g_ phonon dynamics and model both excitation pathways. For the photonic driving case, we calculate the E_g_ phonon evolution **Q**^R^(*t*) by solving the equations of motion (equation (5)) with the driving force defined in equation (6), using the electric field components of the circularly polarized THz field measured by polarization-resolved EOS (Fig. 1e). We focus on the temporal dynamics, rather than the absolute amplitudes, and, therefore, set the Raman tensor coefficient *a* to unity. The damping rate *γ*_R_ used here is identified from the TKE signal and equals 0.22 THz.

To model the phononic case, we solve the system of four equations corresponding to the polarization components of the E_u_ and E_g_ phonon modes $Q=\{{Q}_{x}^{{\rm{IR}}},{Q}_{y}^{{\rm{IR}}},{Q}_{\alpha }^{{\rm{R}}},{Q}_{\beta }^{{\rm{R}}}\}$. Here the IR-active mode is linearly driven by the same circularly polarized THz pulse (Fig. 1e) and coupled with the Raman-active mode via the anharmonic potential *V*(**Q**^IR^, **Q**^R^); therefore, the components of its driving force are defined as ${F}_{i}^{{\rm{IR}}}=Z{E}_{i}^{{\rm{THz}}}-\partial V/\partial {Q}_{i}^{{\rm{IR}}}$. The Born effective charge *Z* and phonon coupling parameter *c* are set to unity for simplicity. The driving force acting on the E_g_ mode is purely defined by the anharmonic coupling potential ${F}_{i}^{{\rm{R}}}=-\partial V/\partial {Q}_{i}^{{\rm{R}}}$. The parameters for the E_u_ phonon mode (*ω*_IR_ = 1.97 THz, *γ*_IR_ = 0.20 THz) are obtained from THz transmission measurements (Methods and Supplementary Text 4) and the E_g_ phonon damping rate is identified by matching the TKE signal decay dynamics (*γ*_R_ = 0.29 THz, corresponding to the phonon lifetime *τ*_R_ = 1/(2π*γ*_R_) = 0.55 ps).

### First-principles calculations

We calculate the phonon eigenfrequencies, eigenvectors, Born effective charges and dielectric function of Bi_2_Se_3_ using the DFT formalism as implemented in Vienna ab initio simulation package (VASP), as well as the frozen-phonon method as implemented in Phonopy. We use projector augmented-wave pseudopotentials with valence electron configurations Bi(6*s*^2^6*p*^3^) and Se(4*s*^2^4*p*^4^), and we use the PBEsol form of the generalized gradient approximation for the exchange–correlation functional. No Hubbard or Hund’s exchange correction is added. We converge the Hellmann–Feynman forces to 10^−5 ^eV Å^−1^ using a plane-wave energy cut-off of 500 eV and an 11 × 11 × 11 *k*-point mesh. The lattice constants of our fully relaxed structure, **a** =4.13 Å and **c** = 28.6 Å, fit reasonably well to experimental values.

To determine the single-mode anharmonicities and nonlinear phonon couplings, we compute the total energy of the system on a 11 × 11 × 11 grid of atomic displacements along the eigenvectors of each of the phonon modes, ${Q}_{{{\rm{E}}}_{{\rm{u}}}^{a}}$, ${Q}_{{{\rm{E}}}_{{\rm{u}}}^{b}}$, ${Q}_{{{\rm{E}}}_{{\rm{g}}}^{a}}$ and ${Q}_{{{\rm{E}}}_{{\rm{g}}}^{b}}$ (Supplementary Fig. 2c,d), and fit the resulting potential energy landscape to the phonon potential energy, *V*. To control the displacement direction, we rotate the mode-effective charge vectors of the E_u_ modes to align them with the *x* and *y* axes. The indices *a* and *b* correspond to this Cartesian system. This rotation mixes the eigenvectors, weighted by the rotation matrix elements, which are then used to generate the displacement grid. For the E_g_ modes, we apply a different rotation such that the resulting Raman tensors correspond to equation (4). To compute the Raman tensors, we determine the static dielectric function from DFT calculations using Vienna ab initio simulation package and evaluate the tensors as derivatives of the dielectric function with respect to the Raman phonon amplitudes (equation (10) in the Supplementary Information). We obtain a value of a Raman tensors’ element *a* = 232 Å^2^/$\sqrt{u}$, where *u* is the atomic mass unit. Further computational details are provided in Supplementary Text 3.

To compute the phonon angular momentum in Fig. 3, we evaluate $$(0,0,{L}_{z}(t))={\bf{Q}}(t)\times \mathop{{\bf{Q}}}\limits^{\cdot }(t)$$, with $${{\bf{Q}}}_{{{\rm{E}}}_{{\rm{u}}}}(t)=({Q}_{{{\rm{E}}}_{{\rm{u}}}^{a}}(t),{Q}_{{{\rm{E}}}_{{\rm{u}}}^{b}}(t),0)$$ and $${{\bf{Q}}}_{{{\rm{E}}}_{{\rm{g}}}}(t)=\left(\right.\!{Q}_{{{\rm{E}}}_{{\rm{g}}}^{a}}(t),$$$${Q}_{{{\rm{E}}}_{{\rm{g}}}^{b}}(t),0\!\left.\right)$$. The mode amplitudes for the phononic pathway are obtained by solving the coupled equations of motion (equations (15)–(18) in the Supplementary Information) using the full anharmonic potential *V* of equation (22) in the Supplementary Information, whereas the photonic pathway is computed from equations (19) and (20) in the Supplementary Information. In both cases, we drive the system with the experimental THz field (Fig. 1e). Real-space atomic trajectories (visualized in Supplementary Fig. 3 and Supplementary Video 3) are reconstructed by projecting the calculated amplitudes $${{\bf{Q}}}_{{{\rm{E}}}_{{\rm{u}}}}(t)$$ and $${{\bf{Q}}}_{{{\rm{E}}}_{{\rm{g}}}}(t)$$ onto the DFT eigenvectors (Supplementary Fig. 2c,d).

### Angular momentum conservation

**Extended Data Fig. 6:**
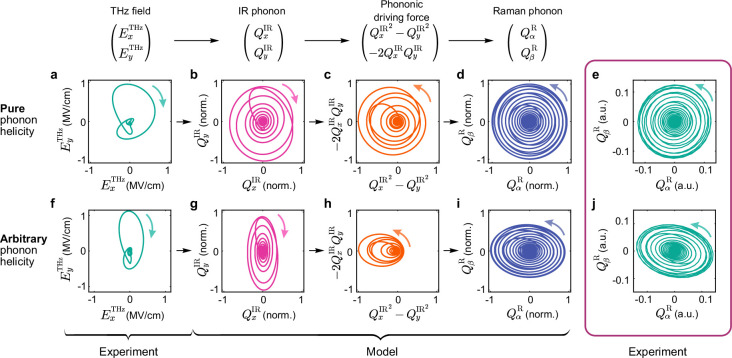
Modeling the phonon trajectory in a classical picture. **a**-**d**, **f**-**i**, Step-by-step modeling of the E_g_ phonon trajectory for purely circular (above) and arbitrary elliptical (below) THz excitation field. **a**, Vectorial trajectory of the right-handed circularly polarized THz electric field, measured by polarization-resolved EOS (Fig. 2a). The arrow indicates the direction of rotation. **b**, Corresponding IR-active E_u_ phonon trajectory, calculated by solving the equation of motion with the driving force set by the experimental THz pulse ${F}_{i}^{{\rm{IR}}}(t)\propto {E}_{i}^{{\rm{THz}}}(t)-\partial V/(\partial {Q}_{i}^{{\rm{IR}}})$, *i* ∈ {*x*, *y*}. **c**, Phononic driving force acting on the Raman-active E_g_ phonon, derived from the symmetry-allowed anharmonic coupling potential $V({{\bf{Q}}}^{{\rm{IR}}},{{\bf{Q}}}^{{\rm{R}}})=c({Q}_{y}^{{{\rm{IR}}}^{2}}-{Q}_{x}^{{{\rm{IR}}}^{2}}){Q}_{\alpha }^{{\rm{R}}}+2c{Q}_{x}^{{\rm{IR}}}{Q}_{y}^{{\rm{IR}}}{Q}_{\beta }^{{\rm{R}}}$ between E_u_ and E_g_ modes as ${F}_{i}^{{\rm{R}}}(t)\propto -\partial V/\partial {Q}_{i}^{{\rm{R}}}$, *i* ∈ *α*, *β*. The driving force exhibits opposite helicity to the IR phonon in **b**. **d**, Calculated trajectory of Raman-active E_g_ phonon. **e**, Experimentally-retrieved E_g_ phonon trajectory, repeated from Fig. 2e. **f**-**j**, Same modeling procedure for arbitrary elliptically polarized THz excitation (Extended Data Fig. 5a), with comparison to the experiment (Extended Data Fig. 5h). Source data

The observed helicity reversal of the E_g_ modes arises from angular momentum conservation between the E_u_ and E_g_ modes in the three-fold crystal lattice, which dictates the form of the driving force. Specifically, the phononic driving force originates from the symmetry-allowed coupling between the IR-active E_u_ mode (transforming as a vector under the *D*_3*d*_ point group) and the Raman-active E_g_ mode (transforming as a symmetric second-rank tensor). Consequently, the driving force acquires the opposite helicity of the E_u_ phonon generating it. This helicity reversal is shown in Fig. 4a–c, where Fig. 4a depicts the trajectory of the E_u_ phonon, and Fig. 4b illustrates the resulting force acting on the E_g_ mode, rotating in the opposite direction and thus carrying an inverted angular momentum. A detailed step-by-step modelling of the E_g_ phonon dynamics, demonstrating the helicity flip, is provided in Extended Data Fig. 6.

The change in the helicity state becomes more evident when transforming from the Cartesian basis to a circular basis $(\widehat{R},\widehat{L})$. In the Cartesian basis, the driving force components are given by

9 $${F}_{\alpha }^{\mathrm{ion}}=c\,\left[{Q}_{x}^{{\mathrm{IR}}^{2}}-{Q}_{y}^{{\mathrm{IR}}^{2}}\right],\,{F}_{\beta }^{\mathrm{ion}}=-2c\,{Q}_{x}^{\mathrm{IR}}{Q}_{y}^{\mathrm{IR}}.$$

To express the force in a circular basis, we rewrite the phonon components and the corresponding driving force as

10 $${Q}_{\left|{\rm{R}}\right\rangle }^{\mathrm{IR}}=\frac{1}{\sqrt{2}}\left[{Q}_{x}^{\mathrm{IR}}+{\rm{i}}{Q}_{y}^{\mathrm{IR}}\right],\,{Q}_{\left|{\rm{L}}\right\rangle }^{\mathrm{IR}}=\frac{1}{\sqrt{2}}\left[{Q}_{x}^{\mathrm{IR}}-{\rm{i}}{Q}_{y}^{\mathrm{IR}}\right],$$

11 $${F}_{\left|R\right\rangle }^{\mathrm{ion}}=\frac{1}{\sqrt{2}}\left[{F}_{\alpha }^{\,\mathrm{ion}}+{\rm{i}}{F}_{\beta }^{\,\mathrm{ion}}\right],\,{F}_{\left|L\right\rangle }^{\,\mathrm{ion}}=\frac{1}{\sqrt{2}}\left[{F}_{\alpha }^{\,\mathrm{ion}}-{\rm{i}}{F}_{\beta }^{\,\mathrm{ion}}\right].$$

Here ${Q}_{\left|R\right\rangle }^{{\rm{IR}}}$ and ${Q}_{\left|L\right\rangle }^{{\rm{IR}}}$ are the RCP and LCP states of the E_u_ phonon, whereas ${F}_{\left|R\right\rangle }^{\,\mathrm{ion}}$ and ${F}_{\left|L\right\rangle }^{\,\mathrm{ion}}$ represent the phononic forces driving the corresponding RCP and LCP states of the E_g_ mode, respectively. In the circular basis, the driving force takes the form

12 $${F}_{\left|R\right\rangle }^{\,\mathrm{ion}}=c\,{Q}_{\left|L\right\rangle }^{{\mathrm{IR}}^{2}},\,{F}_{\left|L\right\rangle }^{\,\mathrm{ion}}=c\,{Q}_{\left|R\right\rangle }^{{\mathrm{IR}}^{2}}.$$

In the circular basis, it becomes clear that the LCP component of the E_u_ phonon drives the RCP E_g_ phonon, and vice versa. This coupling leads to the observed helicity reversal, where the E_g_ mode inherits the opposite helicity of the E_u_ phonon.

## Online content

Any methods, additional references, Nature Portfolio reporting summaries, source data, extended data, supplementary information, acknowledgements, peer review information; details of author contributions and competing interests; and statements of data and code availability are available at 10.1038/s41567-026-03274-8.

## Supplementary information

Supplementary Information Supplementary Texts 1–4, Table 1, Figs. 1–8 and captions for Supplementary Videos 1–3.

Peer Review File

Supplementary Video 1 Experimental data: THz electric field and measured E_g_ phonon trajectories. Animated version of Fig. 1e,f. Trajectory of the THz excitation pulse’s electric field vector (Fig. 1e, left), measured via polarization-resolved EOS, and the corresponding E_g_ phonon trajectory (Fig. 1f, right), measured via the TKE measurement. A shared time reference was obtained from modelling the phononic excitation process (Fig. 3b).

Supplementary Video 2 Simulation: coupled E_u_ phonon and E_g_ phonon trajectories. Animated version of Extended Data Fig. 6b,d. E_u_ phonon trajectory (Extended Data Fig. 6b, left) calculated from the experimental RCP THz excitation field (Fig. 1e) and the corresponding E_g_ phonon trajectory (Extended Data Fig. 6d, right) driven through the lowest-order anharmonic lattice potential $V({{\bf{Q}}}^{{\rm{IR}}},{{\bf{Q}}}^{{\rm{R}}})=c({Q}_{y}^{{{\rm{IR}}}^{2}}-{Q}_{x}^{{{\rm{IR}}}^{2}}){Q}_{\alpha }^{{\rm{R}}}+2c{Q}_{x}^{{\rm{IR}}}{Q}_{y}^{{\rm{IR}}}{Q}_{\beta }^{{\rm{R}}}$.

Supplementary Video 3 Time evolution of phonon helicity reversal based on ab initio DFT. **a**,**b**, Animation shows the time evolution of the atomic displacements for the E_u_ (**a**) and E_g_ (**b**) phonon modes in a Bi_2_Se_3_ unit cell. These dynamics were computed by solving the coupled equations of motion (equations (15)–(18) in the Supplementary Information) with the full anharmonic potential *V* (equations (3) in the Supplementary Information), projected onto eigenvectors derived from ab initio DFT (Supplementary Fig. 2c,d). Phonon amplitudes are independently normalized for clearer visibility. The visualization demonstrates the counter-rotating nature of the interaction: the driven E_u_ mode follows the handedness of the driving field (Fig. 2a, red trace), whereas the induced E_g_ mode rotates in the opposite direction. **c**,**d**, Corresponding quantitative angular momentum dynamics for the E_u_ (**c**) and E_g_ (**d**) modes (Fig. 3c).

## Source data

Source Data Fig. 1 Source data for Fig. 1c–f.

Source Data Fig. 2 Source data for Fig. 2a–d.

Source Data Fig. 3 Source data for Fig. 3b,c.

Source Data Fig. 4 Source data for Fig. 4a–c.

Source Data Extended Data Fig. 1 Source data for Extended Data Fig. 1b–f.

Source Data Extended Data Fig. 2 Source data for Extended Data Fig. 2b–e.

Source Data Extended Data Fig. 3 Source data for Extended Data Fig. 3a–d.

Source Data Extended Data Fig. 4 Source data for Extended Data Fig. 4a–m.

Source Data Extended Data Fig. 5 Source data for Extended Data Fig. 5a–m.

Source Data Extended Data Fig. 6 Source data for Extended Data Fig. 6a–j.

## Data Availability

All data shown in this paper are publicly available via Zenodo (10.5281/zenodo.19087102)^56^. Source data are provided with this paper.
