# Supplementary material for: Observation of angular momentum transfer among crystal lattice modes
Source: Nat Phys. 2026 May 12;22(7):1100–6. doi: 10.1038/s41567-026-03274-8 (PMC13423836; doi:10.1038/s41567-026-03274-8)
Supplement: Supplementary file 1 — Supplementary Texts 1–4, Table 1, Figs. 1–8 and captions for Supplementary Videos 1–3. [file 41567_2026_3274_MOESM1_ESM.pdf]

nature physics

Supplementary information

https://doi.org/10.1038/s41567-026-03274-8

Observation of angular momentum transfer among crystal lattice modes

In the format provided by the authors and unedited

Table of Contents

Supplementary Text

S1. Symmetry analysis and basis convention for E_u and E_g phonon modes S2

S2. Theory of phonon angular momentum upconversion in first-principles calculations S8

S3. Details of first-principle calculations S10

S4. Analytical model for THz transmission measurement of Bi_2Se_3 .... S12

References S15

Supplementary Tables

Table S1. Summary of calculated phonon properties and anharmonic coefficients S11

Supplementary Figures

Figure S1. Vibrational modes of the C_{3v} and D_{3d} model systems S3

Figure S2. Symmetry-adapted eigenvectors and basis handedness S6

Figure S3. Computed real-space atomic trajectories S7

Figure S4. Multilayer thin-film interference model S14

Figure S5. X-ray reflectivity (XRR) S17

Figure S6. X-ray diffraction (XRD) S17

Figure S7. Characterization of y-cut quartz waveplates by THz electro-optic sampling ... S18

Figure S8. Absence of transient magnetic contribution in the Kerr response S19

Supplementary Movies (captions)

Movie S1. Experimental THz electric field and measured E_g phonon trajectories S20

Movie S2. Simulated coupled E_u and E_g phonon trajectories S20

Movie S3. Time evolution of phonon helicity reversal from *ab initio* DFT S20

Supplementary Text

S1. Symmetry analysis and basis convention for E_u and E_g phonon modes

A proper analysis of the coupling symmetry between degenerate phonon modes, particularly their ellipticity states (RCP/LCP), requires an internally consistent basis convention. This is essential for two-dimensional degenerate modes, as their choice of basis is not unique. In the D_{3d} symmetry, the E_g mode is commonly represented by the pair of basis functions $x^2 - y^2$ and $2xy$, although there is a freedom in their relative sign, e.g. $(x^2 - y^2, 2xy)$ vs. $(x^2 - y^2, -2xy)$. While these choices are mathematically equivalent, the sign of the $\pm 2xy$ component determines the handedness of the E_g basis (right- or left-handed), which should be aligned with the handedness of the (x, y) basis of the E_u mode for a clear analysis.

The specific form of the anharmonic coupling potential used in the main text, $V \propto [(Q_x^{\mathrm{IR}})^2 - (Q_y^{\mathrm{IR}})^2]Q_\alpha^{\mathrm{R}} - 2Q_x^{\mathrm{IR}}Q_y^{\mathrm{IR}}Q_\beta^{\mathrm{R}}$, follows directly from the basis convention defined for the atomic displacements in E_u and E_g modes. The sign of the second term is critical; a positive sign (e.g. $+2Q_x^{\mathrm{IR}}Q_y^{\mathrm{IR}}Q_\beta^{\mathrm{R}}$) yields a driving force $\mathbf{F} = (-\partial V/\partial Q_\alpha^{\mathrm{R}}, -\partial V/\partial Q_\beta^{\mathrm{R}}) \propto (Q_x^{\mathrm{IR}\,2} - Q_y^{\mathrm{IR}\,2}, +2Q_x^{\mathrm{IR}}Q_y^{\mathrm{IR}})$ that rotates in the same direction as the E_u mode Q^{IR}, obscuring the helicity reversal observed experimentally and reproduced by DFT. This discrepancy arises from an inconsistent definition of the coordinate systems of IR and Raman modes, where these bases possess opposite handedness. In this section, we demonstrate that preserving a consistent, right-handed coordinate system for both the E_u (x, y) mode and the E_g mode requires the representative E_g basis to transform as $(x^2 - y^2, -2xy)$. This choice establishes the negative sign in the coupling potential, thereby aligning the bases and correctly capturing the counter-rotating driving force reproduced also by DFT calculations.

To resolve the basis ambiguity outlined above, we determine the vibrational eigenvectors of E_u and E_g modes for a model system obeying the D_{3d} symmetry, as in Bi_2Se_3. We restrict the analysis to in-plane displacements, representing the model as two stacked, inverted triangles at $+z$ and $-z$, forming a D_{3d} structure with an inversion center at the origin. The vibrational E_u and E_g eigenvectors of this 6-atom system can be built from the E-symmetry mode of a single C_{3v} triangle (Fig. 4d), extending it to the second triangle based on the mode parity under the inversion i. The E_u mode is odd under inversion, so a displacement (x, y) on the top triangle is matched by an identical (x, y) displacement on its inversion partner. In contrast, the E_g mode is even, therefore a (x, y)

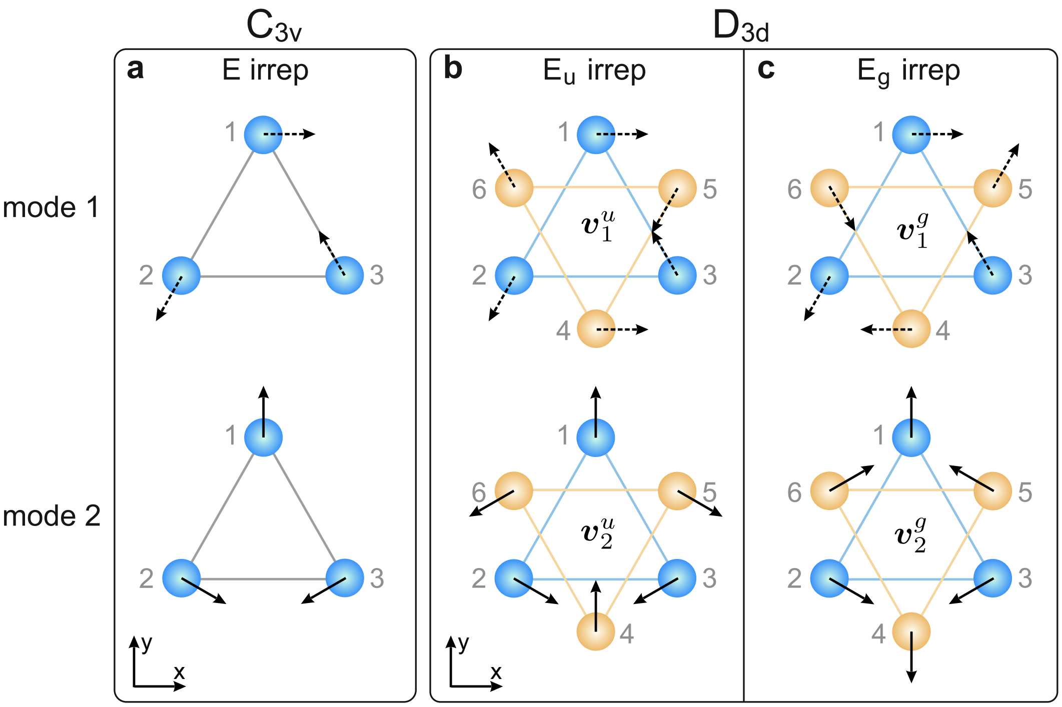

Fig. S1: **Vibrational modes of the C_{3v} and D_{3d} model systems. a**, The doubly degenerate E-symmetry modes of a single triangle C_{3v}. **b-c**, The corresponding E_u and E_g eigenmodes for the D_{3d} system. The E_u modes (**b**) exhibit odd parity under inversion, while the E_g modes (**c**) have even parity. These eigenvectors represent the physical basis obtained using the projection operator method.

displacement on the top atom corresponds to an inverted $(-x, -y)$ displacement on its partner (see Figure S1).

We directly determine these vibrational modes using the projection operator method, a standard technique from group theory. To do this, we describe the system in a 12-dimensional basis representing the (x, y) displacements of the six atoms, $\mathbf{v} = [x_1, y_1, x_2, y_2, \ldots, x_6, y_6]$. Modes belonging to a given irreducible representation (irrep) Γ_j are those that transform according to that irrep, and can be obtained by applying the projection operator $P^{\Gamma_j} = (l_j/h) \sum_R \chi^{(j)}(R)^* D(R)$ to a given vector in the space, extracting the Γ_j component of a vector in the representation space. Here, l_j is the dimension of Γ_j, h is the order of the group, $\chi^{(j)}(R)$ is its character for a symmetry operation R, and $D(R)$ is the 12×12 matrix representation of R, incorporating both the spatial action on (x, y) and the atomic permutation. Diagonalizing P^{Eu} and P^{Eg} yields their eigenvectors $v_i^{u/g}$ with eigenvalue

1, corresponding to the in-plane vibrational modes of the six atoms (Figure S1b,c). These eigenvectors form an arbitrary orthogonal basis spanning each subspace. To straightforwardly connect with mode helicity, we map them onto standard basis functions: the vector basis (x, y) for the $\mathrm{E_u}$ mode and the quadratic basis $(x^2 - y^2, \pm 2xy)$ for the $\mathrm{E_g}$ mode.

To connect the atomic vibrational modes to the mathematical basis, we first analyze their behavior under a symmetry operation that distinguishes each basis component. A convenient choice is the $C_2(x)$ rotation (a π rotation about the x-axis). Under $C_2(x)$, the atoms permute as $1 \leftrightarrow 4, 2 \leftrightarrow 6, 3 \leftrightarrow 5$, while the spatial coordinates transform as $x \longrightarrow x, y \longrightarrow -y$. As a result, x and $x^2 - y^2$ basis functions transform symmetrically (eigenvalue +1), whereas y and $\pm 2xy$ transform antisymmetrically (eigenvalue -1). The representation of the $C_2(x)$ symmetry operation in the 12D space has the form

$$D(C_2) = \begin{pmatrix} \mathbf{0} & \mathbf{0} & \mathbf{0} & \boldsymbol{C}_2 & \mathbf{0} & \mathbf{0} \\ \mathbf{0} & \mathbf{0} & \mathbf{0} & \mathbf{0} & \mathbf{0} & \boldsymbol{C}_2 \\ \mathbf{0} & \mathbf{0} & \mathbf{0} & \mathbf{0} & \boldsymbol{C}_2 & \mathbf{0} \\ \boldsymbol{C}_2 & \mathbf{0} & \mathbf{0} & \mathbf{0} & \mathbf{0} & \mathbf{0} \\ \mathbf{0} & \mathbf{0} & \boldsymbol{C}_2 & \mathbf{0} & \mathbf{0} & \mathbf{0} \\ \mathbf{0} & \boldsymbol{C}_2 & \mathbf{0} & \mathbf{0} & \mathbf{0} & \mathbf{0} \end{pmatrix}, \quad \boldsymbol{C}_2 = \begin{pmatrix} 1 & 0 \\ 0 & -1 \end{pmatrix}, \ \mathbf{0} = \begin{pmatrix} 0 & 0 \\ 0 & 0 \end{pmatrix}. \tag{S1}$$

Here, $\boldsymbol{C}_2$ denotes the 2×2 rotation matrix of $C_2(x)$, $\mathbf{0}$ is the 2×2 zero matrix. The block structure encodes the atomic permutation combined with the vector transformation $\boldsymbol{C}_2$.

When this operator acts on the $\mathrm{E_u}$ and $\mathrm{E_g}$ eigenvectors from Figure S1b,c, the result is

$$\begin{array}{ll} \mathrm{E_u}: & \mathrm{E_g}: \\ D(C_2)\, v_1^u = +1 \cdot v_1^u \quad \longrightarrow v_1^u \sim x & D(C_2)\, v_1^g = -1 \cdot v_1^g \quad \longrightarrow v_1^g \sim 2xy \\ D(C_2)\, v_2^u = -1 \cdot v_2^u \quad \longrightarrow v_2^u \sim y & D(C_2)\, v_2^g = +1 \cdot v_2^g \quad \longrightarrow v_2^g \sim x^2 - y^2 \end{array}$$

The $C_2(x)$ analysis assigns each eigenvector $v_i^{u/g}$ to a symmetric $(x, x^2 - y^2)$ or antisymmetric $(y, 2xy)$ component, leaving their relative sign, and thus the handedness of the coordinate systems, yet undetermined. To resolve this and establish a consistent definition of angular momentum for both modes, we examine their transformation under the C_3 rotation.

We apply $D(C_3)$ (representation of $2\pi/3$ rotation about the z-axis, C_3) to both $\mathrm{E_u}$ $(v_1^u,\ v_2^u)$ and

E_g $(v_2^g,\ v_1^g)$ pairs of eigenvectors. By viewing the outcome of this basis transformation, we will identify a basis convention, where the right- and left-handed polarization is defined the same way for E_u as for E_g modes. The $D(C_3)$ matrix is given by

$$D(C_3) = \begin{pmatrix} \mathbf{0} & \mathbf{0} & \boldsymbol{C}_3 & \mathbf{0} & \mathbf{0} & \mathbf{0} \\ \boldsymbol{C}_3 & \mathbf{0} & \mathbf{0} & \mathbf{0} & \mathbf{0} & \mathbf{0} \\ \mathbf{0} & \boldsymbol{C}_3 & \mathbf{0} & \mathbf{0} & \mathbf{0} & \mathbf{0} \\ \mathbf{0} & \mathbf{0} & \mathbf{0} & \mathbf{0} & \mathbf{0} & \boldsymbol{C}_3 \\ \mathbf{0} & \mathbf{0} & \mathbf{0} & \boldsymbol{C}_3 & \mathbf{0} & \mathbf{0} \\ \mathbf{0} & \mathbf{0} & \mathbf{0} & \mathbf{0} & \boldsymbol{C}_3 & \mathbf{0} \end{pmatrix}, \quad \boldsymbol{C}_3 = \begin{pmatrix} \cos(2\pi/3) & -\sin(2\pi/3) \\ \sin(2\pi/3) & \cos(2\pi/3) \end{pmatrix}, \ \mathbf{0} = \begin{pmatrix} 0 & 0 \\ 0 & 0 \end{pmatrix}. \quad \text{(S2)}$$

The action of $D(C_3)$ on E_u eigenvectors $(v_1^u,\ v_2^u)$ yields

$$D(C_3)\, v_1^u \equiv \cos\left(\frac{2\pi}{3}\right) v_1^u - \sin\left(\frac{2\pi}{3}\right) v_2^u, \quad \text{(S3)}$$

$$D(C_3)\, v_2^u \equiv \sin\left(\frac{2\pi}{3}\right) v_1^u + \cos\left(\frac{2\pi}{3}\right) v_2^u. \quad \text{(S4)}$$

While $D(C_3)$ acts in the full 12-dimensional displacement space, its action within the 2-dimensional E_u subspace reduces to the $+2\pi/3$ rotation of the basis vectors $(v_1^u,\ v_2^u)$. This confirms that the subspace $(v_1^u,\ v_2^u)$ transforms as the vector coordinates (x, y), thereby forming a 'right-handed' basis for the E_u mode. This interpretation is supported by inspecting the atomic displacements in Fig. S1b, where the eigenvector pair $(v_1^u,\ v_2^u)$ forms a right-handed orthogonal basis on each atomic site. Consequently, we establish the following mapping

$$v_1^u \leftrightarrow x, \quad v_2^u \leftrightarrow y.$$

To analyze the E_g eigenvectors $(v_2^g,\ v_1^g)$, we first recall that the associated quadratic basis functions $x^2 - y^2$ and $2xy$ rotate by 2θ, unlike vectors, which rotate by θ. Specifically, under a coordinate rotation of angle $+\theta$, the basis pair transforms as

$$(x^2 - y^2, \pm 2xy) \rightarrow R(\pm 2\theta)(x^2 - y^2, \pm 2xy),$$

where the sign of the second component determines the direction of the induced rotation $R(\pm 2\theta)$. Applying the $D(C_3)$ to the specific eigenvectors yields a rotation of $+4\pi/3$ within the subspace $(v_2^g,\ v_1^g)$

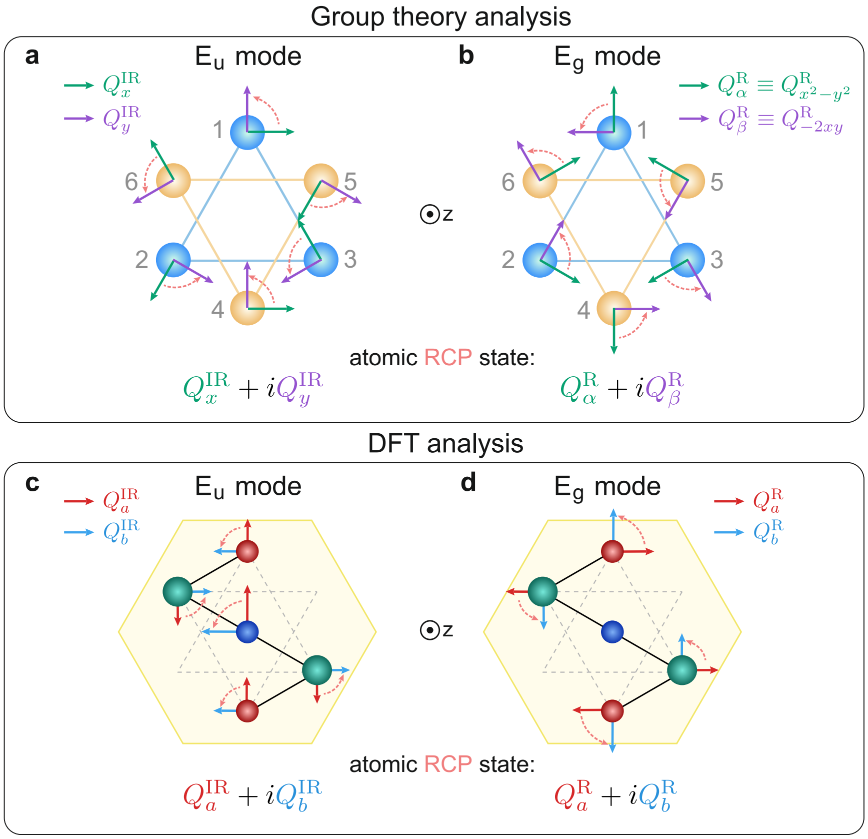

Fig. S2: **Symmetry-adapted eigenvectors and basis handedness. a-b**, Model eigenvectors for $\mathrm{E_u}$ and $\mathrm{E_g}$ modes derived by diagonalizing the projection operators P^{Eu} and P^{Eg}. The $(Q_x^{\mathrm{IR}}, Q_y^{\mathrm{IR}})$ eigenvector pair transforms as (x, y), while $(Q_\alpha^{\mathrm{R}}, Q_\beta^{\mathrm{R}})$ transforms as $(x^2 - y^2, -2xy)$, both forming right-handed orthogonal bases at each atomic site with the z-axis aligned in the same direction. This basis convention enforces the coupling potential to be as Eq.S7 and correctly captures the helicity reversal. **c-d**, Eigenvectors computed from *ab-initio* DFT. These modes exhibit the same local basis orientation as the model, consistent with the derived potential V.

$$D(C_3)\, v_2^g \equiv \cos\left(2 \cdot \frac{2\pi}{3}\right) v_2^g - \sin\left(2 \cdot \frac{2\pi}{3}\right) v_1^g, \tag{S5}$$

$$D(C_3)\, v_1^g \equiv \sin\left(2 \cdot \frac{2\pi}{3}\right) v_2^g + \cos\left(2 \cdot \frac{2\pi}{3}\right) v_1^g. \tag{S6}$$

This rotation of $+2 \cdot 2\pi/3$ identifies the pair with the quadratic basis $(x^2 - y^2, +2xy)$. The result is consistent with the atomic displacements in Fig. S1c, where the pair $(v_2^g,\ v_1^g)$ forms a left-handed orthogonal basis on each atomic site, $v_2^g - i v_1^g$, relative to the right-handed $\mathrm{E_u}$ $(v_1^u,\ v_2^u)$ reference. In a

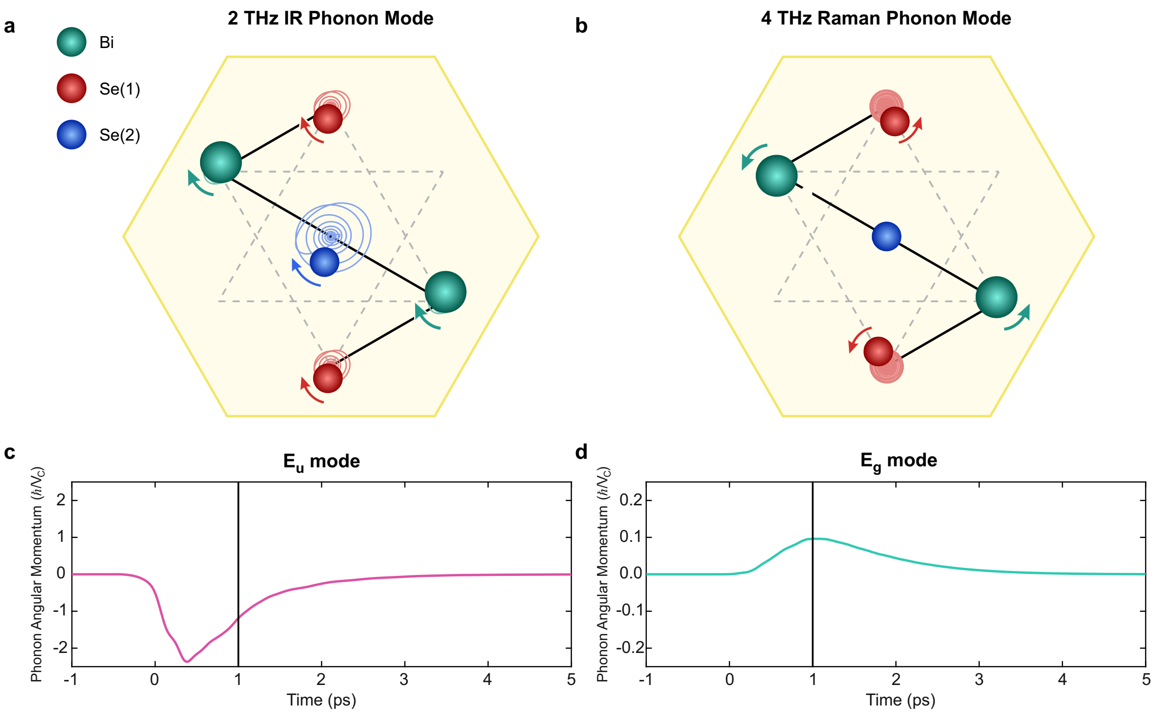

Fig. S3: **Computed real-space atomic trajectories. a-b**, Time-evolution of atomic displacements within the unit cell of Bi_2Se_3, obtained by solving the system of coupled equations of motion (Eqs.S15-S18) with the full form of the coupling potential V (Eq.S22). The solution is projected onto the *ab-initio* eigenvectors shown in Fig.S2c,d. Phonon amplitudes are normalized relative to each other for clearer visibility. The resulting paths visualize the predicted helicity reversal between the driven E_u and the coupled E_g modes (see Supplementary Movie S3). **c-d**, Quantitative *ab-initio* modeling of angular momentum dynamics of E_u and E_g modes (see Fig.3)

three-fold symmetric system, the phase accumulation of $+4\pi/3$ is equivalent to $-2\pi/3$, representing a counter-rotation relative to the E_u reference frame. To enforce a consistent convention, where both bases accumulate the same phase under rotation, we select the basis $(v_2^g, \ -v_1^g)$. This choice maps to $(x^2 - y^2, -2xy)$ and inverts the rotation in the subspace to $-4\pi/3 \equiv +2\pi/3$.

In conclusion, to perform a classical analysis of the symmetry of anharmonic coupling between E_u and E_g phonon modes, the potential must be defined to preserve the same handedness for both

phonon bases

$$V \propto c[(Q_x^{\mathrm{IR}})^2 - (Q_y^{\mathrm{IR}})^2]Q_\alpha^{\mathrm{R}} - 2cQ_x^{\mathrm{IR}}Q_y^{\mathrm{IR}}Q_\beta^{\mathrm{R}}. \tag{S7}$$

This ensures that the circular coordinates, $Q_\alpha^{\mathrm{R}} \pm iQ_\beta^{\mathrm{R}}$ and $Q_x^{\mathrm{IR}} \pm iQ_y^{\mathrm{IR}}$ describe rotations in the same direction. For the *ab-initio* calculations, however, such basis alignment is unnecessary, as the coupling is evaluated directly in the eigenvector basis obtained from DFT. The DFT results independently demonstrate the helicity reversal. To illustrate this, we compute the real-space atomic trajectories by solving the coupled equations of motion (Eqs. S15-S18) with the full coupling potential (Eq.S22), using the *ab-initio* eigenvectors of Fig. S2c,d. The resulting time-dependent displacements are visualized in Fig. S3 and Supplementary Movie S3.

S2. Theory of phonon angular momentum upconversion in first-principle calculations

The equations of motion describing the coherent driving of phonon modes can be expressed as[1]

$$\ddot{Q}_\alpha + \gamma_\alpha \dot{Q}_\alpha + \partial_{Q_\alpha} V = \sum_i Z_{i,\alpha} E_i + \epsilon_0 \sum_{ij} R_{ij,\alpha} E_i E_j, \tag{S8}$$

where Q_α is the phonon amplitude, γ_α is the phonon linewidth, and V is the phonon potential energy. The index $\alpha = \{\mathrm{E}_\mathrm{u}^a, \mathrm{E}_\mathrm{u}^b, \mathrm{E}_\mathrm{g}^a, \mathrm{E}_\mathrm{g}^b\}$ denotes the two orthogonal branches of each of the doubly degenerate $\mathrm{E_u}$ and $\mathrm{E_g}$ modes. We note here that this *ab-initio* theoretical treatment, which is referenced to the crystalline axes (a,b), is connected to the experimental (i.e., labframe coordinates - x,y) description by the relative rotational invariance of the forces, which follows from the rotational symmetry of the system. The right-hand side of the equation contains the light-matter interactions. The first term describes infrared absorption and contains the mode effective charge, $\boldsymbol{Z}_\alpha$, given by

$$\boldsymbol{Z}_\alpha = \sum_n Z_n^* \frac{\boldsymbol{q}_{n,\alpha}}{\sqrt{M_n}}, \tag{S9}$$

where Z_n^* is the Born effective charge tensor of atom n, $\boldsymbol{q}_{n,\alpha}$ is its phonon eigenvector, and M_n its atomic mass, and the sum runs over all atoms in the unit cell. The second term describes Raman scattering and contains the Raman tensors, R_{ij}, given by

$$R_{ij,\alpha} = V_c \frac{\partial \epsilon_{ij}}{\partial Q_\alpha}, \tag{S10}$$

where ϵ_{ij} is the dielectric function, V_c the volume of the unit cell and i, j denote the spatial coordinates. $\boldsymbol{E}(t)$ represents the electric field component of the driving THz pulse, polarized in the

ab plane of the crystal according to the experimental setup. The Raman tensors of the E_g modes, E_g^a and E_g^b, take the following form[2]

$$R_{E_g^a} = \begin{pmatrix} a & 0 \\ 0 & -a \end{pmatrix}, \tag{S11}$$

$$R_{E_g^b} = \begin{pmatrix} 0 & -a \\ -a & 0 \end{pmatrix}. \tag{S12}$$

Because of the symmetry group of the crystal it is possible to choose the coordinates $Q_{E_g^a}$ and $Q_{E_g^b}$ such that they transform, respectively, as $Q^2_{E_u^b} - Q^2_{E_u^a}$ and $Q_{E_u^a} Q_{E_u^b}$. With this choice, the phonon potential energy including anharmonic contributions reads

$$V = \frac{\Omega^2_{E_u}}{2}(Q^2_{E_u^a} + Q^2_{E_u^b}) + \frac{\Omega^2_{E_g}}{2}(Q^2_{E_g^a} + Q^2_{E_g^b}) + cQ_{E_g^a}(Q^2_{E_u^b} - Q^2_{E_u^a}) + 2cQ_{E_g^b} Q_{E_u^a} Q_{E_u^b} + \tilde{V}. \tag{S13}$$

Here, Ω_{E_u} and Ω_{E_g} are the eigenfrequencies of the IR-active E_u and Raman-active E_g modes, respectively, while the three-phonon coupling c denotes the nonlinear phonon coupling of primary interest here. $\tilde{V}$ contains higher order anharmonicities and nonlinear phonon couplings at fourth order, as dictated by the $\bar{3}m$ point-group symmetry,

$$\begin{aligned} \tilde{V} = {} & d_a Q^4_{E_u^a} + d_b Q^4_{E_u^b} + d_c Q^4_{E_g^a} + d_d Q^4_{E_g^b} \\ & + d_{ab} Q^2_{E_u^a} Q^2_{E_u^b} + d_{ac} Q^2_{E_g^a} Q^2_{E_u^a} + d_{bc} Q^2_{E_g^a} Q^2_{E_u^b} + d_{ad} Q^2_{E_g^b} Q^2_{E_u^a} + d_{bd} Q^2_{E_g^b} Q^2_{E_u^b} \\ & + d_{abc} Q^2_{E_g^a} Q_{E_u^a} Q_{E_u^b} + d_{abd} Q^2_{E_g^b} Q_{E_u^a} Q_{E_u^b}. \end{aligned} \tag{S14}$$

Ionic Raman scattering (IRS) is described by three-phonon coupling between two IR-active phonons and one Raman-active phonon, as in Eq. S13. The combined dynamics of the E_u and E_g modes for this process can be obtained by solving the coupled equations of motion resulting from Eq. S8 with $R_{ij} = 0$ and the phonon potential V given by Eq. S13. The equations of motion then read

$$\ddot{Q}_{E_u^a} + \gamma_{E_u} \dot{Q}_{E_u^a} + \Omega^2_{E_u} Q_{E_u^a} = Z_{E_u^a,x} E_x(t) + 2cQ_{E_u^a} Q_{E_g^a} - 2cQ_{E_u^b} Q_{E_g^b} - \partial_{Q_{E_u^a}} \tilde{V}, \tag{S15}$$

$$\ddot{Q}_{E_u^b} + \gamma_{E_u} \dot{Q}_{E_u^b} + \Omega^2_{E_u} Q_{E_u^b} = Z_{E_u^b,y} E_y(t) - 2cQ_{E_u^b} Q_{E_g^a} - 2cQ_{E_u^a} Q_{E_g^b} - \partial_{Q_{E_u^b}} \tilde{V}, \tag{S16}$$

$$\ddot{Q}_{E_g^a} + \gamma_{E_g} \dot{Q}_{E_g^a} + \Omega^2_{E_g} Q_{E_g^a} = -c(Q^2_{E_u^b} - Q^2_{E_u^a}) - \partial_{Q_{E_g^a}} \tilde{V}, \tag{S17}$$

$$\ddot{Q}_{E_g^b} + \gamma_{E_g} \dot{Q}_{E_g^b} + \Omega^2_{E_g} Q_{E_g^b} = -2cQ_{E_u^a} Q_{E_u^b} - \partial_{Q_{E_g^b}} \tilde{V}, \tag{S18}$$

with $\gamma_{\mathrm{E_u}} = 0.2\times 2\pi$ THz·rad and $\gamma_{\mathrm{E_g}} = 0.29\times 2\pi$ THz·rad. In contrast, for terahertz sum-frequency excitation (THz-SFE), we consider a purely harmonic phonon potential, as anharmonicities are disregarded in this process. Thus, only the harmonic terms from Eq. S13 contribute to the equations of motion. Furthermore, in centrosymmetric crystals $\boldsymbol{Z}_\alpha = 0$ for Raman-active phonons, and Eq. S8 accordingly simplifies to

$$\ddot{Q}_{\mathrm{E_g^a}} + \gamma_{\mathrm{E_g}}\dot{Q}_{\mathrm{E_g^a}} + \Omega^2_{\mathrm{E_g}} Q_{\mathrm{E_g^a}} = \epsilon_0 a(E_x^2(t) - E_y^2(t)), \tag{S19}$$

$$\ddot{Q}_{\mathrm{E_g^b}} + \gamma_{\mathrm{E_g}}\dot{Q}_{\mathrm{E_g^b}} + \Omega^2_{\mathrm{E_g}} Q_{\mathrm{E_g^b}} = -2\epsilon_0 a E_x(t)E_y(t). \tag{S20}$$

The two processes differ in the driving force that the coherently excited $\mathrm{E_u}$ modes produce for the Raman-active $\mathrm{E_g}$ modes. In THz-SFE, the driving force is proportional to the square of the electric field (see Eqs. S19 and S20). In contrast, for IRS, the primary component of the driving force is proportional to the square of the IR-active phonon amplitude (see Eqs. S17 and S18).

Finally, the mechanical angular momentum produced by the phonon modes can be obtained in terms of the phonon amplitudes[3]

$$\boldsymbol{L}(t) = \boldsymbol{Q}(t) \times \dot{\boldsymbol{Q}}(t), \tag{S21}$$

where the amplitude vectors are given by $\boldsymbol{Q}_{\mathrm{E_u}}(t) = (Q_{\mathrm{E_u^a}}(t), Q_{\mathrm{E_u^b}}(t), 0)$ and $\boldsymbol{Q}_{\mathrm{E_g}}(t) = (Q_{\mathrm{E_g^a}}(t), Q_{\mathrm{E_g^b}}(t), 0)$, respectively.

S3. Details of first-principle calculations

The general anharmonic phonon potential energy we use for fitting reads

$$\begin{aligned} V = {} & \frac{\Omega^2_{\mathrm{E_u}}}{2}(Q^2_{\mathrm{E_u^a}} + Q^2_{\mathrm{E_u^b}}) + \frac{\Omega^2_{\mathrm{E_g}}}{2}(Q^2_{\mathrm{E_g^a}} + Q^2_{\mathrm{E_g^b}}) \\ & + d_a Q^4_{\mathrm{E_u^a}} + d_b Q^4_{\mathrm{E_u^b}} + d_c Q^4_{\mathrm{E_g^a}} + d_d Q^4_{\mathrm{E_g^b}} \\ & + c_{abc} Q_{\mathrm{E_g^a}} Q_{\mathrm{E_u^a}} Q_{\mathrm{E_u^b}} + c_{abd} Q_{\mathrm{E_g^b}} Q_{\mathrm{E_u^a}} Q_{\mathrm{E_u^b}} \\ & + c_{ac} Q_{\mathrm{E_g^a}} Q^2_{\mathrm{E_u^a}} + c_{ad} Q_{\mathrm{E_g^b}} Q^2_{\mathrm{E_u^a}} + c_{bc} Q_{\mathrm{E_g^a}} Q^2_{\mathrm{E_u^b}} + c_{bd} Q_{\mathrm{E_g^b}} Q^2_{\mathrm{E_u^b}} \\ & + d_{ab} Q^2_{\mathrm{E_u^a}} Q^2_{\mathrm{E_u^b}} + d_{ac} Q^2_{\mathrm{E_g^a}} Q^2_{\mathrm{E_u^a}} + d_{ad} Q^2_{\mathrm{E_g^b}} Q^2_{\mathrm{E_u^a}} + d_{bc} Q^2_{\mathrm{E_g^a}} Q^2_{\mathrm{E_u^b}} + d_{bd} Q^2_{\mathrm{E_g^b}} Q^2_{\mathrm{E_u^b}} \\ & + d_{abc} Q^2_{\mathrm{E_g^a}} Q_{\mathrm{E_u^a}} Q_{\mathrm{E_u^b}} + d_{abd} Q^2_{\mathrm{E_g^b}} Q_{\mathrm{E_u^a}} Q_{\mathrm{E_u^b}}. \end{aligned} \tag{S22}$$

This general potential with mixed components of the doubly degenerate modes can be brought back into the form of Eq. S13 with a coordinate transformation. Next, we compute the total energy

Table S1: **Summary of calculated phonon properties and anharmonic coefficients.** Calculated eigenfrequencies in THz, single-mode anharmonicities and nonlinear phonon couplings in meV/(Å$\sqrt{u}$)^n, n being the order of the phonon amplitude and u the atomic mass unit, and mode effective charges in $e/\sqrt{u}$, where e is the elementary charge.

	c_{abc}	c_{abd}	c_{ac}	c_{ad}	c_{bc}	c_{bd}	d_a	d_b	d_c	d_d	d_{ab}	d_{ac}	d_{ad}	d_{bc}	d_{bd}	d_{abc}	d_{abd}	Z
E_u^a(2.6)							0.3				0.5							1.04
E_u^b(2.6)								0.3			0.5							1.04
E_g^a(4.2)	-1.1		1.3		-1.3				-0.02			-0.02		0.2		0.07		0
E_g^b(4.2)		-2.6		-0.6		0.6				-0.02			0.2		-0.02		-0.07	0

on a $11 \times 11 \times 11$ grid defined by atomic displacements along the eigenvectors of the rotated E_u and E_g modes. The resulting anharmonic potential explicitly includes coupling terms between the doubly degenerate E_u modes and each of the E_g modes separately, while no coupling is considered between the individual E_g modes themselves, which is deemed to be small.

We perform a three-step fitting procedure on the resulting potential energy landscape to extract single-mode anharmonicities and nonlinear phonon couplings from the phonon potential energy V in Eq. S22. First, we obtain the single-mode anharmonicities for the E_u and E_g modes by fitting the potential energy landscape for each mode individually. We use the total energy from the grid points where displacements occur along a single-phonon mode, with the others fixed to zero. From this, we obtain the coefficients $\Omega_{E_u}, \Omega_{E_g}, d_a, d_b, d_c, d_d$. We include the phonon frequencies in the fitting to ensure that they match those computed with PHONOPY. Next, we focus on the term that involved only the E_u modes, $Q^2_{E_u^a} Q^2_{E_u^b}$, by fitting the potential energy landscape to the total energy values where only the E_u modes are displaced, with the E_g modes held at zero. The single-mode anharmonicities of the first step are kept fixed, allowing us to extract the coefficient d_{ab}. Finally, we fit the potential energy landscape to the terms in V (in Eq. S22) that involve the three phonon modes for each of the E_g modes, using total energy values from the grid points where all modes are displaced. With the coefficients from the previous steps held constant, we determine the nonlinear phonon couplings d_{ac}, d_{ad}, d_{bc}, d_{bd},d_{abc}, d_{abd}. Table S1 in Supplementary Information summarizes the computed phonon properties and anharmonic coefficients.

We note that the calculated phonon frequencies are off by about 30 % and 5% for the E_u and E_g modes, respectively, with respect to experimental values. This discrepancy is known to arise in

DFT calculations for this class of material[4,5]. Accordingly, also the nonlinear couplings could be varying by two-digit percentages compared to experiment. Such a variation would lead to small quantitative changes in the calculated angular momentum, but would, however, leave the analysis and interpretation of the mechanisms unchanged. Additionally, as pointed out in[4], temperature can influence phonon eigenfrequencies. Therefore, part of the discrepancy between the DFT and experimental results likely arises from the fact that the DFT calculations are performed at zero temperature, whereas the experiments are conducted at room temperature.

S4. Analytical model for THz transmission measurement of Bi_2Se_3

To analyze the THz transmittance of Bi_2Se_3 (Extended Data Fig. 1d) and determine the phonon lifetime of the E_u mode τ_{IR}, we derive the analytical expression based on Fresnel coefficients. The analytical solution accounts for all internal reflections within both the Bi_2Se_3 sample and the sapphire substrate. Owing to the topological insulating properties of Bi_2Se_3, the material is represented as a three-layer system comprising an insulating bulk sandwiched between two conductive topological surface states (TSS)[6,7]. Figure S4 illustrates the layered structure and defines the associated transmission and reflection coefficients. The multilayer stack consists of a TSS (n_1, refractive index), a Bi_2Se_3 bulk layer (n_2, refractive index), a TSS (n_1, refractive index), and a sapphire substrate (n_3, refractive index), surrounded by air (n_0, refractive index) from both sides. The THz pulse propagates at normal incidence through the layers in the sequence $n_0 \rightarrow n_1 \rightarrow n_2 \rightarrow n_1 \rightarrow n_3 \rightarrow n_0$.

The reference transmission function for sapphire, $T_{\text{sapph}}(\omega)$, is derived for a single layer, accounting for multiple beam interference within the substrate

$$T_{\text{sapph}}(\omega) = \frac{t_{03}t_{30}e^{i\varphi_3}}{1 - r_{30}^2 e^{i2\varphi_3}}. \tag{S23}$$

Here, $t_{ij}(\omega)$ and $r_{ij}(\omega)$ denote the Fresnel transmission and reflection coefficients for a wave propagating from media i to j, defined as $t_{ij}(\omega) = 2n_i/(n_i + n_j)$ and $r_{ij}(\omega) = (n_i - n_j)/(n_i + n_j)$. The phase φ_3 accounts for the THz pulse propagation through the sapphire layer of thickness $d_3 = 500\ \mu\text{m}$, given by $\varphi_3(\omega) = (\omega n_3(\omega) d_3)/c_o$, where c_o is the speed of light in vacuum.

To derive the sample transmission function $T_{\text{total}}(\omega)$, we employ an iterative approach that systematically reduces a multilayer system to an effective single-layer model by incorporating

the contribution of other layers into the modified transmission and reflection coefficients at the interfaces. As the first step, the Bi_2Se_3 sample is treated as an effective interface between air and the sapphire substrate, characterized by the transmission (T_{03}) and reflection (R_{30}) coefficients. This simplification reduces the system to an equivalent single-layer sapphire (n_3) model, analogous to the reference case, so the THz transmission function through the sample and the substrate $T_{\text{total}}(\omega)$ can be expressed as

$$T_{\text{total}}(\omega) = \frac{T_{03} t_{30} e^{i\varphi_3}}{1 - r_{30} R_{30} e^{i2\varphi_3}}. \tag{S24}$$

To determine T_{03} and R_{30}, we separately consider a three-layer Bi_2Se_3 sample and reduce its complexity to a single layer-model of the bulk (n_2) sandwiched between air (n_0) and sapphire (n_3), with effective interfaces defined by the TSSs (Fig. S4a). Here we introduce the transmission and reflection coefficients at the air-bulk (T_{02}, R_{20}), and bulk-sapphire (T_{23}, R_{23}, T_{32}, R_{32}) interfaces, so the transmission and reflection of the Bi_2Se_3 sample can be written as:

$$T_{03}(\omega) = \frac{T_{02} T_{23} e^{i\varphi_2}}{1 - R_{20} R_{23} e^{i2\varphi_2}}, \quad R_{30}(\omega) = R_{32} + \frac{T_{32} T_{23} R_{20} e^{i2\varphi_2}}{1 - R_{20} R_{23} e^{i2\varphi_2}}, \tag{S25}$$

where $\varphi_2(\omega) = (\omega n_2(\omega) d_2)/c_o$ stands for the phase accumulated by the THz pulse propagating through the Bi_2Se_3 bulk with a thickness of $d_2 = 0.3$ μm. The frequency-dependent coefficients $T_{02}(\omega)$, $R_{20}(\omega)$ and $T_{23}(\omega)$, $R_{23}(\omega)$, $T_{32}(\omega)$, $R_{32}(\omega)$ are explicitly derived by considering two TSS layers independently: one interfacing with air and the bulk, and the other with the bulk and sapphire (see Fig. S4b,c). These coefficients can be expressed in term of the Fresnel coefficients as follows

$$T_{02}(\omega) = \frac{t_{01} t_{12} e^{i\varphi_1}}{1 - r_{10} r_{12} e^{i2\varphi_1}}, \quad R_{20}(\omega) = r_{21} + \frac{t_{21} t_{12} r_{10} e^{i2\varphi_1}}{1 - r_{10} r_{12} e^{i2\varphi_1}}, \tag{S26}$$

$$T_{23}(\omega) = \frac{t_{21} t_{13} e^{i\varphi_1}}{1 - r_{13} r_{12} e^{i2\varphi_1}}, \quad R_{23}(\omega) = r_{21} + \frac{t_{21} t_{12} r_{13} e^{i2\varphi_1}}{1 - r_{13} r_{12} e^{i2\varphi_1}}, \tag{S27}$$

$$T_{32}(\omega) = \frac{t_{31} t_{12} e^{i\varphi_1}}{1 - r_{13} r_{12} e^{i2\varphi_1}}, \quad R_{32}(\omega) = r_{31} + \frac{t_{31} t_{13} r_{12} e^{i2\varphi_1}}{1 - r_{13} r_{12} e^{i2\varphi_1}}, \tag{S28}$$

where $\varphi_1(\omega) = (\omega n_1(\omega) d_1)/c_o$ with $d_1 = 1$ nm.

Therefore, using this iterative approach, we reduce the model's complexity and derive the THz transmission function $T_{\text{total}}(\omega)$ in terms of only Fresnel coefficients and phase factors. The calculated THz transmission function of Bi_2Se_3, presented in Extended Data Fig. 1d, is determined by

$$T_{Bi_2Se_3}(\omega) = T_{\text{total}}(\omega)/T_{\text{sapph}}(\omega). \tag{S29}$$

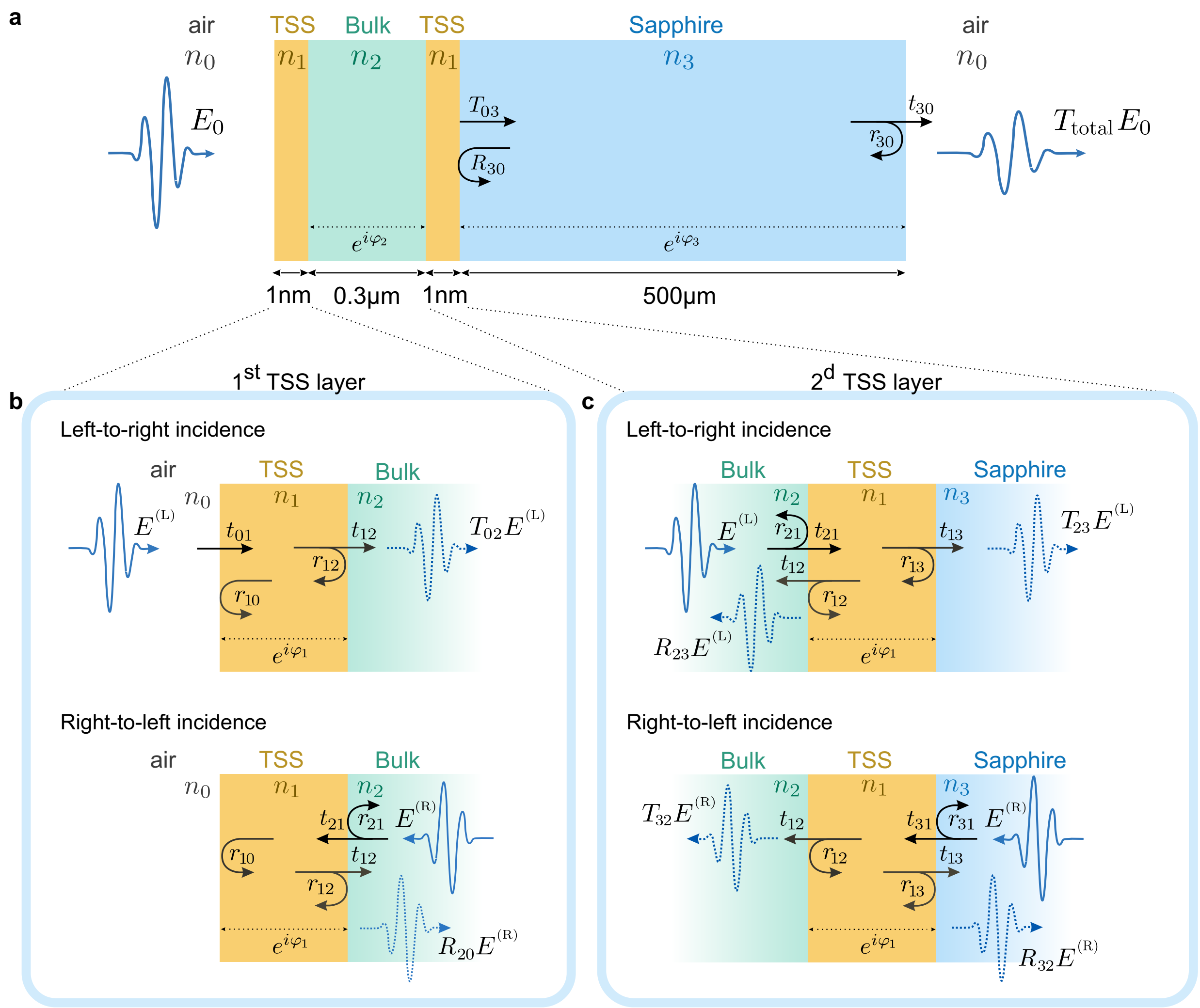

Fig. S4: **Multilayer thin-film interference model. a**, Schematic of multilayer system of Bi_2Se_3 sample on the sapphire substrate and the transmitted THz field (blue waveform). Bi_2Se_3 is represented as a three-layer system of an insulating bulk layer sandwiched between two conductive surface layers (TSS). **b-c**, Transmission and reflection of the THz pulse from the first and second TSS layers, respectively. t_{ij} and r_{ij} are the Fresnel transmission and reflection coefficients for a wave propagating from medium i into medium j. T_{ij} and R_{ij} accounts for all internal reflections between the media i and j.

To model the Bi_2Se_3 measured transmittance obtained from the windowed time-domain EOS traces (see Extended Data Fig. 1f), we keep only the contribution from the fundamental transmitted THz pulse by retaining only the numerators in $T_{\text{total}}(\omega)$ and $T_{\text{sapph}}(\omega)$. The sample and substrate transmission functions therefore take the form

$$T_{\text{total}}(\omega) = T_{03} t_{30} e^{i\varphi_3}, \tag{S30}$$

$$T_{\text{sapph}}(\omega) = t_{03} t_{30} e^{i\varphi_3}, \tag{S31}$$

respectively, leading to the Bi_2Se_3 transmission function $T_{\text{Bi}_2\text{Se}_3}(\omega) = T_{03}(\omega)/t_{03}(\omega)$.

The dielectric functions for the distinct layers of the Bi_2Se_3 sample are defined using different models tailored to their properties. The Lorentz oscillator model is employed for the insulating Bi_2Se_3 bulk $\varepsilon_{\text{bulk}}(\omega)$, while the Drude model is used for the conducting topological surface states $\varepsilon_{\text{TSS}}(\omega)$. The dielectric functions for air and sapphire are set as $\varepsilon_{\text{air}}(\omega) = 1$ and $\varepsilon_{\text{sapph}}(\omega) = 3$. For the topological surface states, the dielectric function is given by

$$\varepsilon_{\text{TSS}}(\omega) = 1 - \frac{\omega_{\text{pl}}^2}{\omega^2 + i\gamma\omega}, \tag{S32}$$

with ω_{pl}=1050 THz and $\gamma = 20$ THz. The introduction of conducting TSS layers at the interfaces allows to capture the reduction in the THz transmission across the spectrum. For the Bi_2Se_3 bulk, the dielectric function is set as

$$\varepsilon_{\text{bulk}}(\omega) = \varepsilon_\infty + \frac{S\omega_{\text{IR}}^2}{\omega_{\text{IR}}^2 - \omega^2 - i\gamma_{\text{IR}}\omega}, \tag{S33}$$

with $\varepsilon_\infty = 40$, $S = 100.5$, $\omega_{\text{IR}} = 1.97$ THz and $\gamma_{\text{IR}} = 0.20$ THz. The parameters ω_{IR}, γ_{IR}, S are the frequency of IR-active E_u phonon, its damping rate and the oscillator strength, respectively. These parameters of the dielectric functions were adjusted to model the experimental data, focusing on determining the phonon frequency ω_{IR} and phonon damping rate γ_{IR}. The E_u phonon lifetime is determined as $\tau_{\text{IR}} = 1/(2\pi\gamma_{\text{IR}})$ and equals 0.79 ps.

References

[1] Juraschek, D. M. & Maehrlein, S. F. Sum-frequency ionic Raman scattering. *Physical Review B* **97**, 174302 (2018).

[2] Loudon, R. The Raman effect in crystals. *Advances in Physics* **13**, 423–482 (1964).

[3] Juraschek, D. M., Fechner, M., Balatsky, A. V. & Spaldin, N. A. Dynamical multiferroicity. *Physical Review Materials* **1**, 014401 (2017).

[4] Wang, Q. H., Kalantar-Zadeh, K., Kis, A., Coleman, J. N. & Strano, M. S. Electronics and optoelectronics of two-dimensional transition metal dichalcogenides. *Nature nanotechnology* **7**, 699–712 (2012).

[5] Boulares, I. *et al.* Surface phonons in the topological insulators Bi_2Se_3 and Bi_2Te_3. *Solid State Communications* **271**, 1–5 (2018).

[6] Chen, S. *et al.* Real-space nanoimaging of THz polaritons in the topological insulator Bi_2Se_3. *Nature Communications* **13**, 1374 (2022).

[7] Pogna, E. A. A. *et al.* Mapping propagation of collective modes in Bi_2Se_3 and $Bi_2Te_{2.2}Se_{0.8}$ topological insulators by near-field terahertz nanoscopy. *Nature Communications* **12**, 6672 (2021).

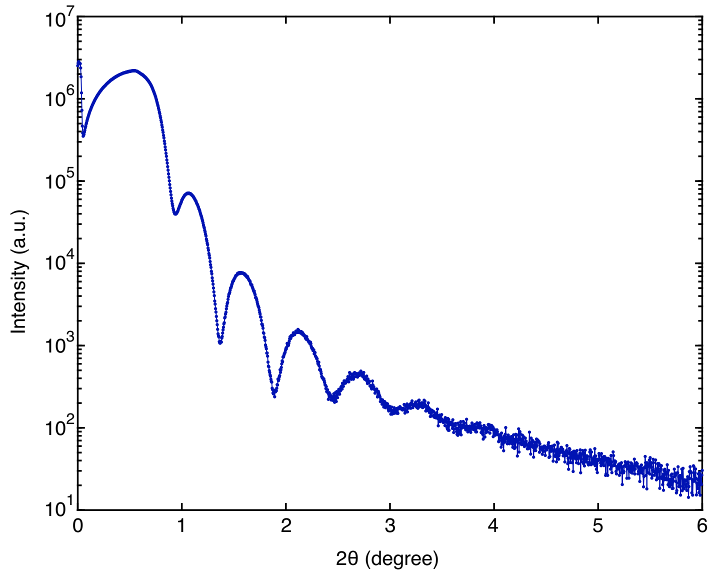

Fig. S5: **X-ray reflectivity (XRR).** XRR curve of the used Bi_2Se_3 sample. From the periodicity of the oscillations, a Bi_2Se_3 thickness d =15.0 nm is determined.

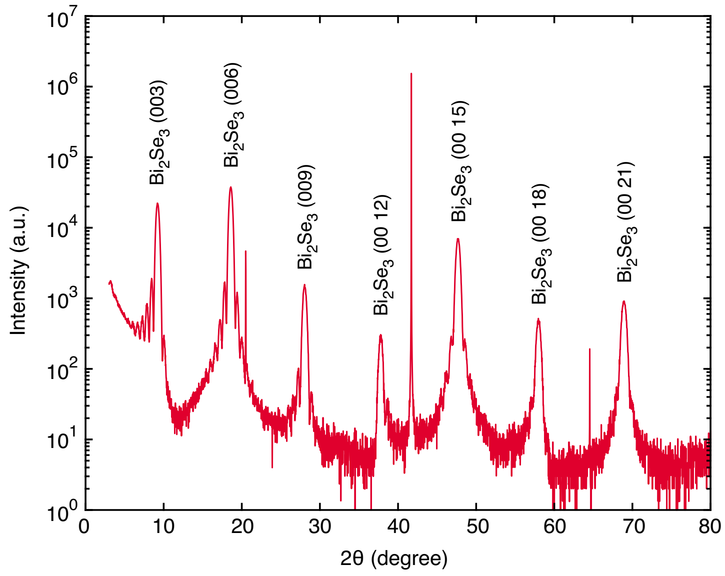

Fig. S6: **X-ray Diffraction (XRD).** Symmetric $2\theta/\theta$ XRD curve of the investigated sample with numerous peaks from the epilayer, evidencing the correct Bi_2Se_3 stoichiometry as well as the single-crystal nature of the Bi_2Se_3 film.

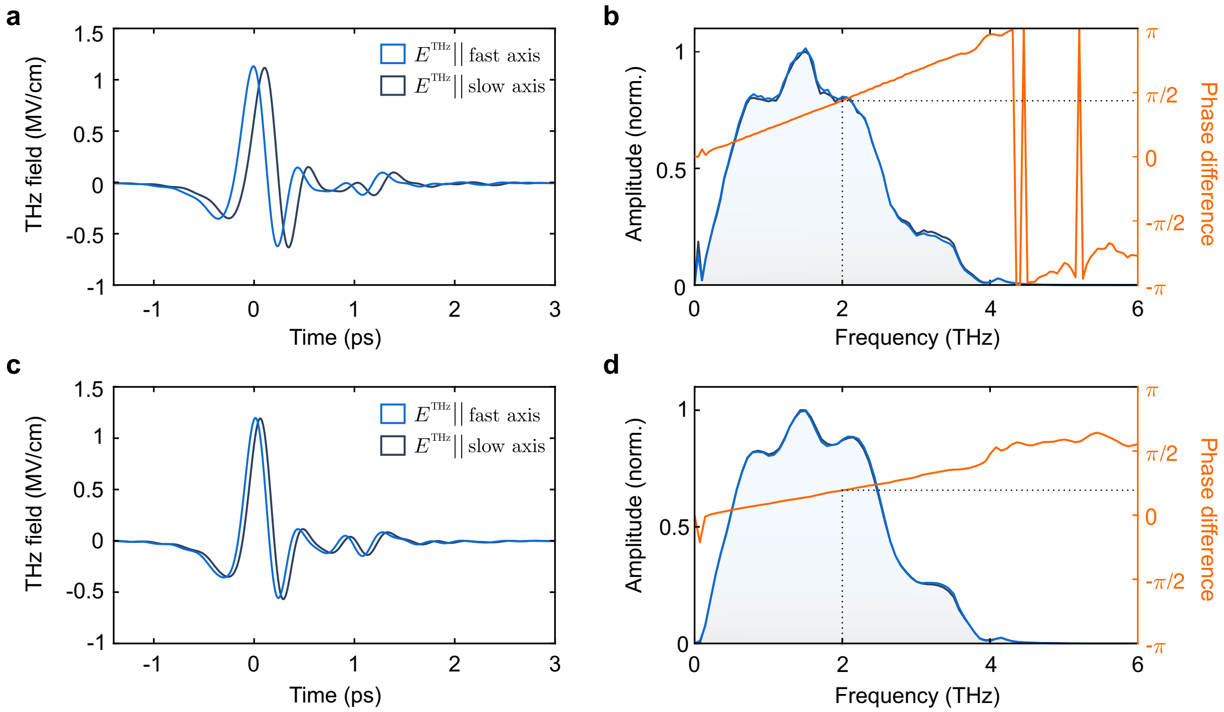

Fig. S7: **Characterization of y-cut quartz waveplates by THz electro-optic sampling. a**, Linearly-polarized THz electric fields after propagating through a 700 μm-thick y-cut quartz waveplate, measured via electro-optic sampling in a 50 μm-thick z-cut quartz detection crystal. Blue and black traces correspond to THz fields aligned with the fast and slow axes of y-cut quartz, respectively. **b**, Corresponding Fourier transforms of the THz fields and their relative phase difference (orange). At 2 THz, the phase difference is about $\pi/2$, indicating the generation of a circularly polarized THz pulse when the incident field is oriented at 45° to the fast and slow axes. **c-d**, Same measurements as **a–b**, but for a 380 μm-thick y-cut quartz waveplate. The phase difference at 2 THz results in the generation of an arbitrarily elliptical THz polarization state when the incident THz field is oriented at 45° relative to the crystal axes.

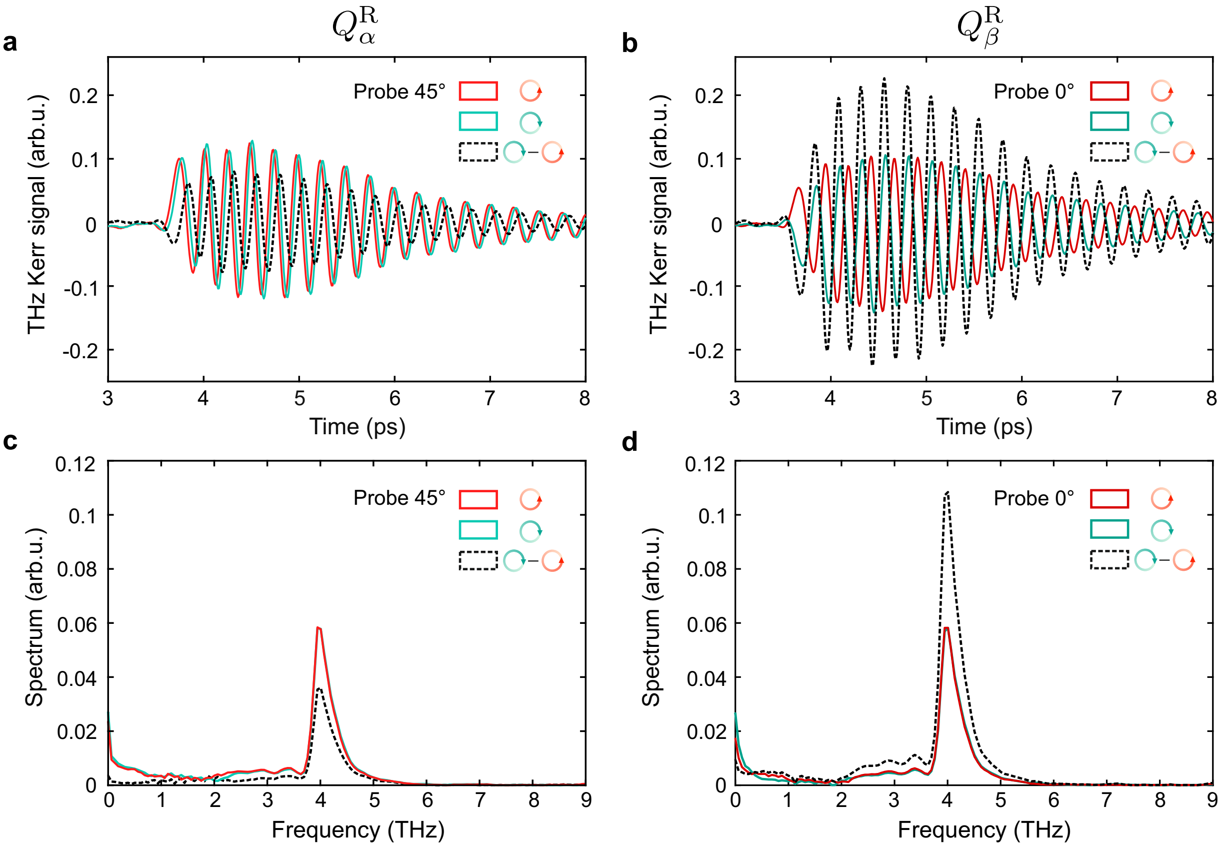

Fig. S8: **Absence of transient magnetic contribution in the Kerr response.** THz Kerr signals measured under right- (red) and left- (blue) circularly polarized THz excitation for probe polarizations of 45° and 0° (data presented in Extended Data Fig.4f,i) and their difference (dashed black) in the time (panels **a**,**b**) and frequency (panels **c**,**d**) domains. A pump-probe time drift in the RCP and LCP measurements introduces a small temporal shift between nearly identical Kerr traces. For 45° probe polarization, RCP and LCP THz pulses drive the Q_α^{R} component with the same phase, but the temporal shift causes the subtraction to produce a residual oscillation of the same shape. For 0° probe, the opposite phases of the driven Q_β^{R} component lead the subtraction to enhance the oscillation. In both cases, the RCP–LCP difference shows no helicity-dependent slowly-varying background, confirming the absence of any magnetically induced Kerr contribution on the order of the phonon coherence time.

Caption for Movie S1. Experimental data: THz electric field and measured E_g phonon trajectories Animated version of Figure 1e,f. Trajectory of the THz excitation pulse's electric field vector (left; Fig. 1e), measured via polarization-resolved electro-optic sampling, and the corresponding E_g phonon trajectory (right; Fig. 1f), measured via the THz-induced Kerr effect measurement. A shared time reference was obtained from modeling the phononic excitation process (Fig. 3b).

Caption for Movie S2. Simulation: Coupled E_u phonon and E_g phonon trajectories. Animated version of Extended Data Fig. 6b,d. E_u phonon trajectory (left; Extended Data Fig. 6b) calculated from the experimental RCP THz excitation field (Fig. 1e) and the corresponding E_g phonon trajectory (right; Extended Data Fig. 6d) driven through the lowest-order anharmonic lattice potential $V(\boldsymbol{Q}^{\mathrm{IR}}, \boldsymbol{Q}^{\mathrm{R}}) = c[(Q_y^{\mathrm{IR}})^2 - (Q_x^{\mathrm{IR}})^2]Q_\alpha^{\mathrm{R}} + 2c\, Q_x^{\mathrm{IR}} Q_y^{\mathrm{IR}} Q_\beta^{\mathrm{R}}$.

Caption for Movie S3. Time evolution of phonon helicity reversal based on ab-initio DFT. **a**-**b**, The animation displays the time evolution of the atomic displacements for the E_u (**a**) and E_g (**b**) phonon modes in Bi_2Se_3 unit cell. These dynamics were computed by solving the coupled equations of motion (Eqs. S15–S18) with the full anharmonic potential V (Eq. S22), projected onto eigenvectors derived from *ab-initio* DFT (Fig. S2c,d). Phonon amplitudes are independently normalized for clearer visibility. The visualization demonstrates the counter-rotating nature of the interaction: the driven E_u mode follows the handedness of the driving field (Fig. 2a, red trace), while the induced E_g mode rotates in the opposite direction. **c**-**d**, Corresponding quantitative angular momentum dynamics for the E_u (**c**) and E_g (**d**) modes (presented in Fig.3c).
