# Supplementary material for: Observation of angular momentum transfer among crystal lattice modes
Source: Nat Phys. 2026 May 12;22(7):1100–6. doi: 10.1038/s41567-026-03274-8 (PMC13423836; doi:10.1038/s41567-026-03274-8)
Supplement: Supplementary file 2 — Peer Review File [file 41567_2026_3274_MOESM2_ESM.pdf]

nature portfolio

Peer Review File

Observation of angular momentum transfer among crystal lattice modes

Corresponding Author: Professor Sebastian Maehrlein

This file contains all reviewer reports in order by version, followed by all author rebuttals in order by version.

Attachments originally included by the reviewers as part of their assessment can be found at the end of this file.

Version 0:

Reviewer comments:

Reviewer #1

(Remarks to the Author)
The paper addresses one of the key questions of non-equilibrium magnetism, namely, how does the angular momentum get transferred between the phonon modes after the initial excitation and spin-lattice relaxation.

For the first time, the authors *directly* observe the transfer of angular momentum from two IR-active phonon modes into one Raman-active phonon mode. In contrast to other approaches, the angular momentum of the resulting mode is measured directly, through the non-magnetic THz-induced Kerr effect, which paves the way for future detailed studies of angular momentum dynamics in and spin relaxation in solids.

Importantly, the authors performed ab-initio modelling that allowed to distinguish the signal from a three-phonon process from a two-photon excitation of the Raman-active phonon.

The main result of the paper - that phonon angular momentum transfer takes place via lattice anharmonicity and conserves angular momentum - is of key importance for magnetism, both from the fundamental perspective as well as for future applications. The manuscript is well-written and should be accessible to a broad audience of physicists.

Therefore I recommend the paper to be accepted for publication in Nature Physics.

Reviewer #2

(Remarks to the Author)
The topic of chiral phonons, and in particular how angular momentum can be transferred from such phonons to spins, has become an extremely interesting topic as of late. This has been highlighted by multiple high-impact articles published in recent years e.g. Refs. [4,10-12,17-18,20], to name but a few. In the majority of these articles, angular momentum flow from phonons to the spins has been traced using magneto-optical techniques, which is somewhat indirect. In this article, Minakova et al propose to use ionic Raman scattering to directly follow the flow of angular momentum from the targeted infrared-active phonon to Raman-active ones. This is an important precursor for any studies aiming to use chiral phonons to deliver angular momentum to other sub-systems. I therefore find the article highly interesting, and believe it will also be interesting to a broad range of physicists.

To achieve the above, Minakova et al aim to measure the trajectory of the coupled Raman Eg mode using non-magnetic Kerr effect. The signals shown in Fig. 1 are extremely impressive. Following the THz excitation, the resulting THz Kerr signal displays a a clear pi/2 phase-shift with the probe polarization oriented at 0deg or 45deg. Despite the clear non-circular polarization of the impinging pulse (panel e), which is probably inescapable due to the single-cycle nature of the excitation, the phonon trajectory (panel f) is circular beyond any doubt.

Going further, the authors even demonstrate that the helicity of the incident THz pulse is opposite to the helicity of the circular Raman phonon mode. This can be explained by the nonlinear phonon-phonon coupling, with the switching of helicity being given by conservation of angular momentum. The authors provide convincing measurements showing that

ellipticity can even be transferred efficiently. All this is accompanied by ab-initio and semi-classical modelling.

I find this an extremely insightful and rich manuscript, which experimentally proves that circularly-polarized infrared-active Eu phonons can transfer angular momentum to Eg phonons. In my opinion, this result definitely warrants publication in Nature Physics, as it bridges an important gap in our understanding that has been overlooked until now.

I have a few questions for the authors, which I hope they will be able to resolve.
1) The authors choose to study Bi2Se3 because it has Raman-active and IR-active doubly-degenerate phonons at 4.0 THz and 2.0 THz. For the coupling observed in this paper to occur, is it therefore compulsory for the Raman-active phonon to have a frequency twice that of the IR-active phonon? In other words, if the ratio of the resonances was e.g. 2.5:1, and the THz pulse was rather narrow-band, would the observed effect not emerge? I think it would be good to highlight this point. In complex materials with many IR-active and Raman-active phonon modes, the observed TKE stemming from broadband excitations could always emerge to some degree, complicating the analysis.

2) The resonant excitation of doubly-degenerate Eu phonon modes lead to the creation of a transient magnetization, which would (I expect) be measured in terms of a magneto-optical polarization rotation. This would equally result in a polarization rotation as obtained through the balanced detection scheme (e.g. as used in Ref [17]). How do the authors exclude any possibility of transient magnetization? I suppose that any magnetic signal would result in a rectified signal that is independent of the probe polarization orientation, but perhaps the authors can clarify this better.

3) In Fig. 3, the authors mention that 3% of the Eu angular momentum is transferred to the Eg mode. Why is this so low? And how could the amount of angular momentum be in principle increased?

4) The authors could also include a citation for https://arxiv.org/abs/2507.22232. This is a relevant work which fits well in e.g. line 59.

Reviewer #3

(Remarks to the Author)
see attached file

Version 1:

Reviewer comments:

Reviewer #2

(Remarks to the Author)
I have carefully read the response of the authors to the comments, particularly in relation to Referee 3. I believe the authors responded well, and I find all their extra analysis and interpretation convincing. The manuscript has certainly improved as a result. I recommend publication in Nature Physics as-is.

Reviewer #3

(Remarks to the Author)
My comments have been addressed. I recommend publication as is.

Open Access This Peer Review File is licensed under a Creative Commons Attribution 4.0 International License, which permits use, sharing, adaptation, distribution and reproduction in any medium or format, as long as you give appropriate credit to the original author(s) and the source, provide a link to the Creative Commons license, and indicate if changes were made.
In cases where reviewers are anonymous, credit should be given to 'Anonymous Referee' and the source.
The images or other third party material in this Peer Review File are included in the article's Creative Commons license, unless indicated otherwise in a credit line to the material. If material is not included in the article's Creative Commons license and your intended use is not permitted by statutory regulation or exceeds the permitted use, you will need to obtain permission directly from the copyright holder.
To view a copy of this license, visit https://creativecommons.org/licenses/by/4.0/

General remarks

We thank all three reviewers for their thorough evaluation of our work, as well as for their very positive and expert feedback. Their comments and suggestions have clearly helped us to improve the manuscript and to clarify the last unclear points.

Please find our detailed responses below, along with the corresponding revisions of the main manuscript and Supplementary Information.

In general:

- Responses to the reviewers below are given in blue font and the corresponding actions are highlighted in green font.
- In the highlighted documents (main manuscript & Supplementary Information), all changes are given in green font, and specified here by line numbers of the highlighted documents.

Response to Reviewer #1

The paper addresses one of the key questions of non-equilibrium magnetism, namely, how does the angular momentum get transferred between the phonon modes after the initial excitation and spin-lattice relaxation.

For the first time, the authors directly observe the transfer of angular momentum from two IR-active phonon modes into one Raman-active phonon mode. In contrast to other approaches, the angular momentum of the resulting mode is measured directly, through the non-magnetic THz-induced Kerr effect, which paves the way for future detailed studies of angular momentum dynamics in and spin relaxation in solids.

Importantly, the authors performed ab-initio modelling that allowed to distinguish the signal from a three-phonon process from a two-photon excitation of the Raman-active phonon.

The main result of the paper - that phonon angular momentum transfer takes place via lattice anharmonicity and conserves angular momentum - is of key importance for magnetism, both from the fundamental perspective as well as for future applications. The manuscript is well-written and should be accessible to a broad audience of physicists.

Therefore I recommend the paper to be accepted for publication in Nature Physics.

We thank Reviewer #1 for their outstanding assessment of our work and for highlighting its fundamental importance to the field.

Response to Reviewer #2

The topic of chiral phonons, and in particular how angular momentum can be transferred from such phonons to spins, has become an extremely interesting topic as of late. This has been highlighted by multiple high-impact articles published in recent years e.g. Refs. [4,10-12,17-18,20], to name but a few. In the majority of these articles, angular momentum flow from phonons to the spins has been traced using magneto-optical techniques, which is somewhat indirect. In this article, Minakova et al propose to use ionic Raman scattering to directly follow the flow of angular momentum from the targeted infrared-active phonon to Raman-active ones. This is an important precursor for any studies aiming to use chiral phonons to deliver angular momentum to other sub-systems. I therefore find the article highly interesting, and believe it will also be interesting to a broad range of physicists.

To achieve the above, Minakova et al aim to measure the trajectory of the coupled Raman Eg mode using non-magnetic Kerr effect. The signals shown in Fig. 1 are extremely impressive. Following the THz excitation, the resulting THz Kerr signal displays a clear pi/2 phase-shift with the probe polarization oriented at 0deg or 45deg. Despite the clear non-circular polarization of the impinging pulse (panel e), which is probably inescapable due to the single-cycle nature of the excitation, the phonon trajectory (panel f) is circular beyond any doubt.

Going further, the authors even demonstrate that the helicity of the incident THz pulse is opposite to the helicity of the circular Raman phonon mode. This can be explained by the nonlinear phonon-phonon coupling, with the switching of helicity being given by conservation of angular momentum. The authors provide convincing measurements showing that ellipticity can even be transferred efficiently. All this is accompanied by ab-initio and semi-classical modelling.

I find this an extremely insightful and rich manuscript, which experimentally proves that circularly-polarized infrared-active Eu phonons can transfer angular momentum to Eg phonons. In my opinion, this result definitely warrants publication in Nature Physics, as it bridges an important gap in our understanding that has been overlooked until now.

We thank Reviewer #2 for their very positive evaluation of the manuscript and their constructive feedback.

I have a few questions for the authors, which I hope they will be able to resolve.

1) The authors choose to study Bi2Se3 because it has Raman-active and IR-active doubly-degenerate phonons at 4.0 THz and 2.0 THz. For the coupling observed in this paper to occur, is it therefore compulsory for the Raman-active phonon to have a frequency twice that of

the IR-active phonon? In other words, if the ratio of the resonances was e.g. 2.5:1, and the THz pulse was rather narrow-band, would the observed effect not emerge? I think it would be good to highlight this point. In complex materials with many IR-active and Raman-active phonon modes, the observed TKE stemming from broadband excitations could always emerge to some degree, complicating the analysis.

Reply 2.1

We thank the reviewer for raising this important point. The frequency doubling condition is not totally strict, but the efficiency of the coupling is indeed maximized under $\Omega_{\mathrm{R}} = 2\Omega_{\mathrm{IR}}$ condition. To be more precise, the efficiency of the coupling depends on the spectral overlap between the Raman mode and the corresponding driving force generated by the IR-active mode, $F(t) \propto Q_{\mathrm{IR}}^2(t)$. In the frequency domain, the driving force $Q_{\mathrm{IR}}^2(t)$ results in a convolution $F(\omega) \propto \{Q_{\mathrm{IR}} * Q_{\mathrm{IR}}\}(\omega)$ of two Lorenzians $Q_{\mathrm{IR}}(\omega)$ both centered at Ω_{IR} with linewidth γ_{IR}. The resulting driving force F is also a Lorenzian centered at $2\Omega_{\mathrm{IR}}$ with linewidth $2\gamma_{\mathrm{IR}}$. Therefore, the Raman-active mode can be efficiently driven via the $Q_{\mathrm{R}}Q_{\mathrm{IR}}^2$ coupling term if its frequency Ω_{R} lies within the range $|\Omega_{\mathrm{R}} - 2\Omega_{\mathrm{IR}}| \lesssim \gamma_{\mathrm{R}} + 2\gamma_{\mathrm{IR}}$. If a narrowband THz field still creates a sufficient amplitude of the IR-active phonon, the angular momentum transfer is still equally efficient, as the sum-frequency ionic Raman scattering does not rely on impulsiveness[1].

In Bi_2Se_3, the 2 THz IR-active phonon mode has a damping constant of γ_{IR}=0.2 THz, resulting in a driving force with 0.4 THz spectral linewidth. The restriction to the frequency of Raman active phonon for the effective anharmonic coupling is $|\Omega_{\mathrm{R}} - 2\Omega_{\mathrm{IR}}| \lesssim \gamma_{\mathrm{R}} + 2\gamma_{\mathrm{IR}} = 0.29 + 0.4$ THz = 0.69 THz, therefore the ratio of the resonances in the range of 1.7:1 to 2.3:1 is still sufficient for phonon mode to be effectively coupled. If this frequency matching condition is not satisfied, the higher-order terms (see Eq.13 in Methods) can, in principle, mediate the Raman phonon excitation, albeit with weaker efficiency.

We also fully agree with the reviewer that complex materials with many IR- and Raman-active modes will on one hand complicate the exact mode assignment, but on the other hand possibly deliver more options for anharmonic phonon-phonon coupling and thus provide more angular momentum transfer channels. In this case, the exact angular momentum transfer channel can be disentangled via 2D THz excitation[2].

Actions:

- We added a brief section on the spectral overlap conditions to the **Methods** under "Excitation pathways of the E_g mode" (**lines 422-427**)
- and refer to it in the **Main** manuscript at **line 83**.

2) The resonant excitation of doubly-degenerate Eu phonon modes lead to the creation of a transient magnetization, which would (I expect) be measured in terms of a magneto-optical polarization rotation. This would equally result in a polarization rotation as obtained through the balanced detection scheme (e.g. as used in Ref [17]). How do the authors exclude any possibility of transient magnetization? I suppose that any magnetic signal would result in a rectified signal that is independent of the probe polarization orientation, but perhaps the authors can clarify this better.

Reply 2.2

We fully agree with Reviewer #2 that potential magnetic contributions to the measured Kerr signal should be clarified. In Bi_2Se_3, we believe such a mechanism can be excluded on material and experimental grounds. In paramagnetic systems such as CeF_3, as mentioned by the reviewer[3], circularly polarized E_u phonons can induce a transient spin polarization via phonon-spin coupling leading to a significant helicity-dependent Kerr response. In contrast, Bi_2Se_3 does not exhibit ions with unpaired spins and is thus intrinsically diamagnetic. All of our measurements were performed at room temperature, above the regime where any paramagnetic response could emerge. Consistently, our measured Kerr rotation shows no helicity dependent, slowly varying unipolar background (see Extended Data Fig.4f,i). An instantaneous electronic TKE response scaling with E_{THz}^2 appears only for linearly polarized THz excitation (see Extended Data Fig.4c). Under circular excitation, the rotating field averages out the anisotropic component of E_{THz}^2, suppressing the electronic contribution.

Transient magneto-optical Kerr effects have also been observed in diamagnetic materials under circular THz excitation, such as $SrTiO_3$[4] and $LiNbO_3$[5], unveiling dynamical multiferroicity and chiral phonon-polaritons, respectively. In those cases, a clear indicator of transient magnetization is a helicity-dependent, rectified component of the Kerr signals. In our measurements in Bi_2Se_3, the RCP-LCP difference yields no such rectified feature (see new Figure S8 below). In case of 45° probe

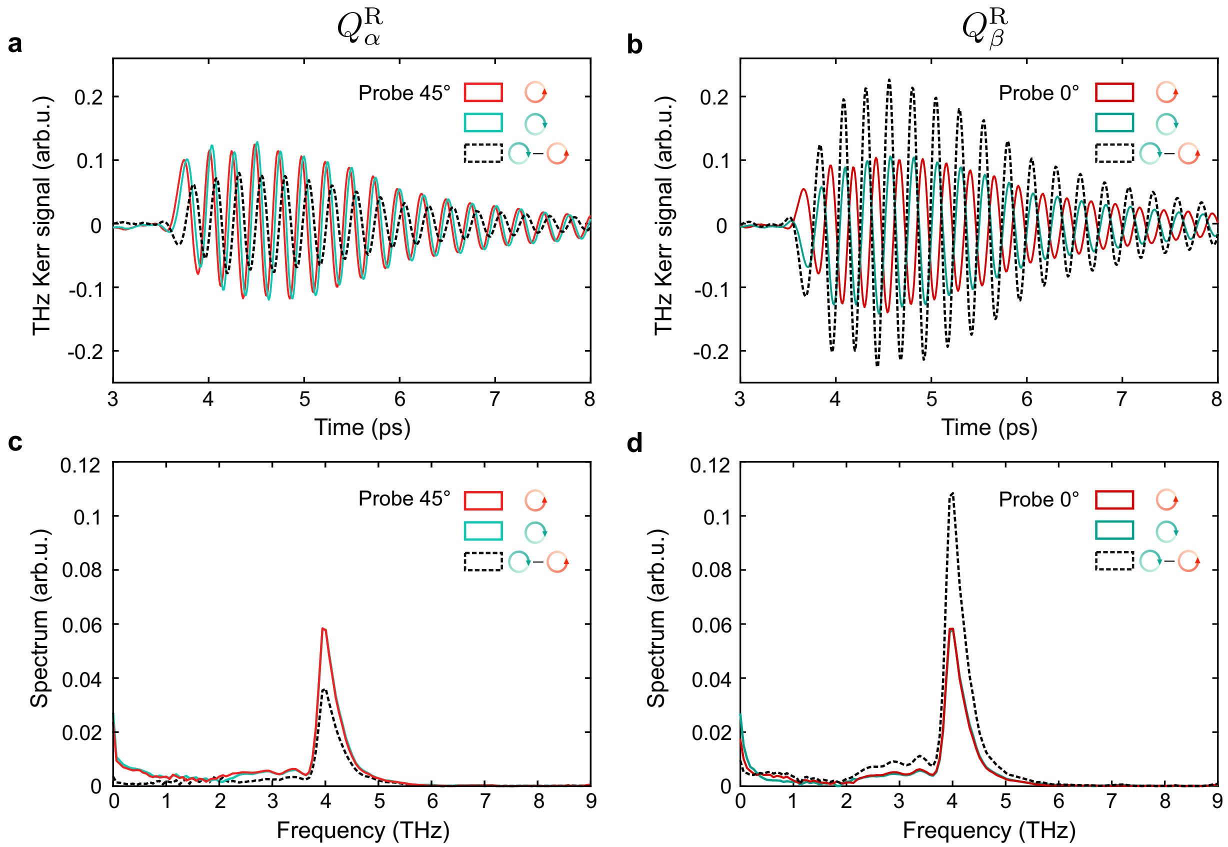

Fig. S8: **Absence of transient magnetic contribution in Kerr response.** THz Kerr signals measured under right- (red) and left- (blue) circularly polarized THz excitation for probe polarizations of 45° and 0° (data presented in Extended Data Fig.4f,i) and their difference (dashed black) in the time (panels **a**,**b**) and frequency (panels **c**,**d**) domains. A pump-probe time drift in the RCP and LCP measurements introduces a small temporal shift between nearly identical Kerr traces. For a 45° probe polarization, RCP and LCP THz pulses drive the Q_α^{R} component with the same phase, but the timing shift causes the subtraction to produce a residual oscillation of the same shape. For a 0° probe, the opposite phases of the driven Q_β^{R} component lead the subtraction to enhance the oscillation. In both cases, the RCP–LCP difference shows no helicity-dependent rectified background, confirming the absence of any magnetically induced Kerr contribution.

polarization, RCP and LCP THz excitation induce the same phase of the $\mathrm{E_g}$ component Q_α^{R} and the difference RCP–LCP thus vanishes. The residual RCP–LCP signal in Fig. S8a,c is due to a small drift in pump-probe delay between RCP and LCP measurements. In case of 0° probe polarization, the RCP and LCP excitations drive the Q_β^{R} component of the $\mathrm{E_g}$ mode with opposite phase and thus

the subtraction doubles the oscillatory response, which again does not show a rectified component. The unchanged spectral profiles Fig. S8c,d between the original traces and their difference indicates that the Kerr rotation contains no apparent contribution from transient magnetization.

Actions:

- We now mention the absence of any magnetic Kerr contribution in the **Main** text (**line 109**).
- We also added the new **Figure S8** to the **Supplementary Information** and refer to it in the **Main** text.

3) In Fig. 3, the authors mention that 3% of the Eu angular momentum is transferred to the Eg mode. Why is this so low? And how could the amount of angular momentum be in principle increased?

Reply 2.3

The amount of angular momentum transferred to the E_g mode primarily depends on three variables: (1) the nonlinear coupling coefficient between the E_u and E_g modes, (2) the frequency matching condition for the sum-frequency excitation mechanism (see **Reply 1.1** above), and (3) the total damping of the phonon modes. Increasing (1) and (2) will increase the percentage of angular momentum transferred to the E_g mode, while increasing (3) will decrease it. The rest of the angular momentum goes into the acoustic phonon continuum, which we do not capture explicitly, as we use a phenomenological damping coefficient for both phonon modes.

Actions:

- In the revised **Main** manuscript, we briefly comment on the dependencies of the amount of up-converted angular momentum in **lines 190-192**.
- There, we also mention the phenomenological treatment of the angular momentum loss to the lower-energy acoustic phonon continuum (see **lines 191-192**).

4) The authors could also include a citation for https://arxiv.org/abs/2507.22232. This is a relevant work which fits well in e.g. line 59.

Reply 2.4

We apologize for not having updated our earlier references to include this recent, very relevant preprint article. In the revised version of the manuscript, of course, we updated our bibliography with this important contribution dealing with transient magnetic fields of axial or chiral phonons in dielectric materials.

Actions: We added the reference to the work by Biggs et al. to **lines 54 and 64**.

Response to Reviewer #3

First of all, we want to generally thank Reviewer #3 for their in-depth expert review and for keeping an eye on the credibility of the whole research field. This perspective is deeply appreciated by us. In the following, we will reply step by step to each of Reviewer #3's comments.

The mathematics underlying the results in this paper is fairly simple. For the anharmonic interaction $V = V_0\left[(Q_x^2 - Q_y^2)Q_\alpha + 2Q_xQ_yQ_\beta\right]$, *the equations of motion are*

$$\ddot{Q}_\alpha + \Omega_R^2 Q_\alpha = -V_0(Q_x^2 - Q_y^2), \qquad \ddot{Q}_\beta + \Omega_R^2 Q_\beta = -2V_0 Q_x Q_y.$$

Here, the two components of the polar and Raman modes are labeled (Q_x, Q_y) *and* (Q_α, Q_β), *respectively. For* $Q_x = A\sin\omega t$ *and* $Q_y = A\cos\omega t$, *we get*

$$Q_\alpha = +\frac{A^2V_0}{\Omega_R^2 - 4\omega^2}\cos 2\omega t \qquad Q_\beta = -\frac{A^2V_0}{\Omega_R^2 - 4\omega^2}\sin 2\omega t.$$

Thus, if the coherent E_u *mode is, say, left handed, the coherently driven* E_g *mode is right handed and vice versa. The experimental results are in very good agreement with this analysis, and the evidence that the driving mechanism for the* E_g *phonon is lattice anharmonicity (rather than electron–phonon interaction) is strong. The remaining question is: what, if anything, does this have to do with angular momentum transfer, as claimed by the authors?*

Reply 3.A

We thank the reviewer for highlighting the good agreement of our experimental results with the fundamental symmetry analysis and for confirming the strong evidence of the presented driving mechanism via lattice anharmonicity. Reviewer #3 is absolutely right in raising the central question of how angular momentum should be interpreted in this context. We agree that the distinction

between "real" angular momentum (associated with continuous rotational symmetry) and pseudo angular momentum (or crystal angular momentum, associated with discrete rotational symmetry) is subtle and essential here. Motivated by the reviewer's question, we revisited the problem at a deeper microscopic level, focusing explicitly on the atomic-scale motion underlying the coherently driven E_u and E_g phonon modes. In this regard, we found it useful to analyze the dynamics from complementary viewpoints, group symmetry considerations and first-principles calculations, which together clarify the presence of circular ionic motion and the associated helicity reversal, dictated by the discrete three-fold rotational symmetry of the crystal. This perspective allows us to address the question of "real" angular momentum transfer and its relation to pseudo angular momentum conservation, as this is a justified recurring point in Reviewer #3's comments:

1) We fully agree that "real" angular momentum is rigorously defined only in the presence of continuous rotational symmetry (e.g., $SO(3)$ for orbital angular momentum or $SU(2)$ for spin). In a crystal with discrete rotational symmetry such as D_{3d} in Bi_2Se_3, angular momentum is therefore not a generally conserved quantity, and the usual free-space definitions cannot be directly applied at the level of the lattice symmetry.

However, for the coherently driven doubly degenerate zone-center E_u and E_g modes, the ionic displacements at each atomic site follow circular trajectories with a well-defined handedness with respect to a fixed lab frame (see **Reply 3.B** below) independent of the crystal's azimuthal orientation around its c-axis. According to Streib[6], this provides the external lab reference frame to measure "real" ("Newtonian") angular momentum. Each ion thus carries a local microscopic angular momentum given by $\boldsymbol{u} \times \dot{\boldsymbol{u}}$ about its equilibrium position. Because all ions participating in a given coherent mode rotate in the same sense, these microscopic angular momenta add up to a net mode angular momentum, even though this quantity is not protected by a continuous rotational symmetry. This is the microscopic "real" angular momentum we refer to in the manuscript.

2) We also agree with the reviewer that our experimental results are completely expected from fundamental symmetry considerations. Nevertheless, our work is the **first experimental verification** of this universal concept, dictating the relation between discrete rotational invariance of a solid and the pseudo angular momentum conservation among its crystalline lattice modes. We thereby provide the experimental reasoning for the analogy between the established concept of

crystal momentum (pseudo linear momentum) and crystal angular momentum (pseudo angular momentum).

3) In the revised manuscript, we now provide two complementary approaches to show that the coherent phonon states excited and observed in this work a) carry microscopic **"real" angular momentum** and b) are coupled via a helicity reversal in real space (see **Reply 3.B** below concerning the axis of reference). The latter would violate the angular momentum conservation in free space, but perfectly obeys the **pseudo angular momentum conservation** due to the discrete C_3 symmetry of the investigated material.

Approach 1: Point group symmetry and basis convention for E_u and E_g phonon modes (new **Supplementary Text S1** and **Figs. S1, S2**)

We extended the simplified picture of an E mode in the C_{3v} point group[7] (shown in main Fig. 4d-f) to the inversion symmetric D_{3d} point group of Bi_2Se_3. In the new Supplementary Text S1, we demonstrate that the E_u and E_g modes' eigenvectors of the model system, chosen to transform as (x, y) and $(x^2 - y^2, -2xy)$ respectively, form an orthogonal right-handed basis at each atomic site. This definition ensures that even though the E_g modes transforms as quadratic functions, a circular excitation of the E_g state results in well-defined circular motion at the atomic level. Furthermore, this consistent basis choice confirms the correct relative signs in the quadratic basis, $-2xy$ (instead of $2xy$) with respect to $x^2 - y^2$, and provides the anharmonic coupling

$$V \propto (Q_x^{\mathrm{IR}^2} - Q_y^{\mathrm{IR}^2})Q_\alpha^{\mathrm{R}} - 2Q_x^{\mathrm{IR}}Q_y^{\mathrm{IR}}Q_\beta^{\mathrm{R}}, \tag{1}$$

which dictates the helicity reversal between the two coupled motions in real space (see Figure S2 below). If we therefore induce a "real" microscopic angular momentum by a coherent preparation of $Q_x^{\mathrm{IR}}\dot{Q}_y^{\mathrm{IR}} - Q_y^{\mathrm{IR}}\dot{Q}_x^{\mathrm{IR}}$ (effectively taking real spin angular momentum from the circularly polarized THz light field by absorption), the anharmonic coupling leads to a "real" microscopic angular momentum with opposite sign of the z-component in the E_g mode. This reversal of "real" angular momentum, nevertheless conserves pseudo angular momentum and thus demonstrates that "real" angular momentum is not a well-defined observable under discrete rotational invariance. In our view, this provides an instructive analogy to the phonon-phonon Umklapp process, which conserves

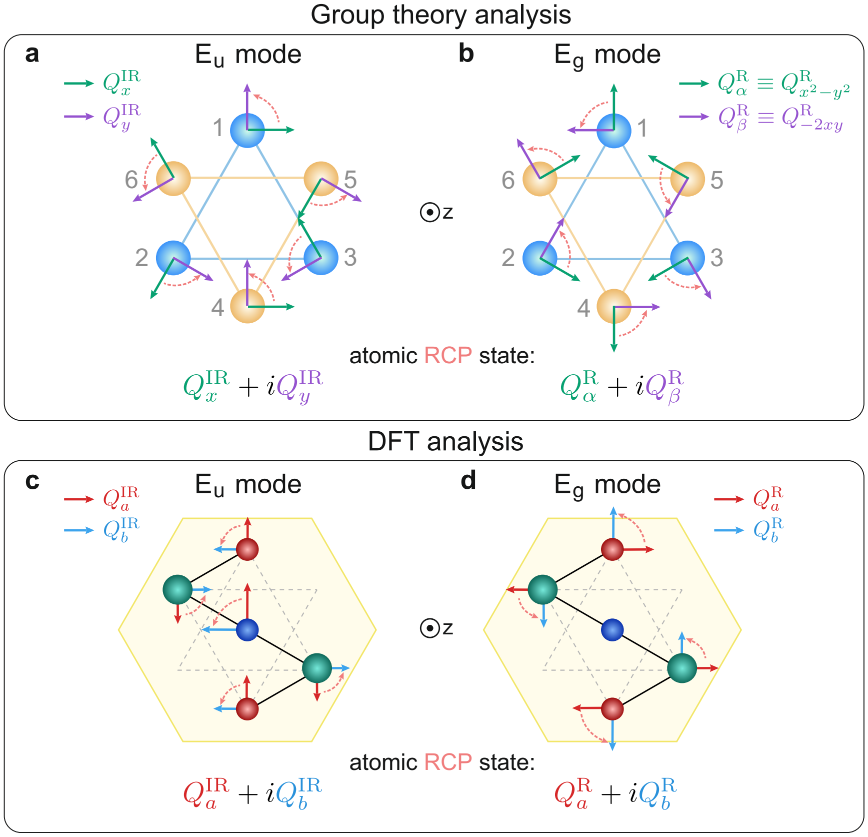

Fig. S2: **Symmetry-adapted eigenvectors and basis handedness. a-b**, Model eigenvectors for $\mathrm{E_u}$ and $\mathrm{E_g}$ modes derived by diagonalizing the projection operators P^{Eu} and P^{Eg}. The $(Q_x^{\mathrm{IR}}, Q_y^{\mathrm{IR}})$ eigenvector pair transforms as (x, y), while $(Q_\alpha^{\mathrm{R}}, Q_\beta^{\mathrm{R}})$ transforms as $(x^2 - y^2, -2xy)$, both forming right-handed orthogonal bases at each atomic site with the z-axis aligned in the same direction. This basis convention enforces the coupling potential to be as Eq.7 and correctly captures the helicity reversal. **c-d**, Eigenvectors computed from *ab-initio* DFT. These modes exhibit the same local basis orientation as the model, consistent with the derived potential V.

pseudo linear momentum by reversing its sign (see details **Reply 3.2** below).

Approach 2: Mode-resolved real space ion trajectories from first-principles calculations (new **Supplementary Information Fig. S3** and **Supplementary Movie S3**):

For a more instructive view on our DFT-based modelling employing the experimentally measured THz fields (summarized in Fig. 3c), we plotted and animated the resulting real space ion trajectories for each mode in Fig. S3 and Supplementary Movie S3, respectively. For both modes ($\mathrm{E_u}$ and $\mathrm{E_g}$),

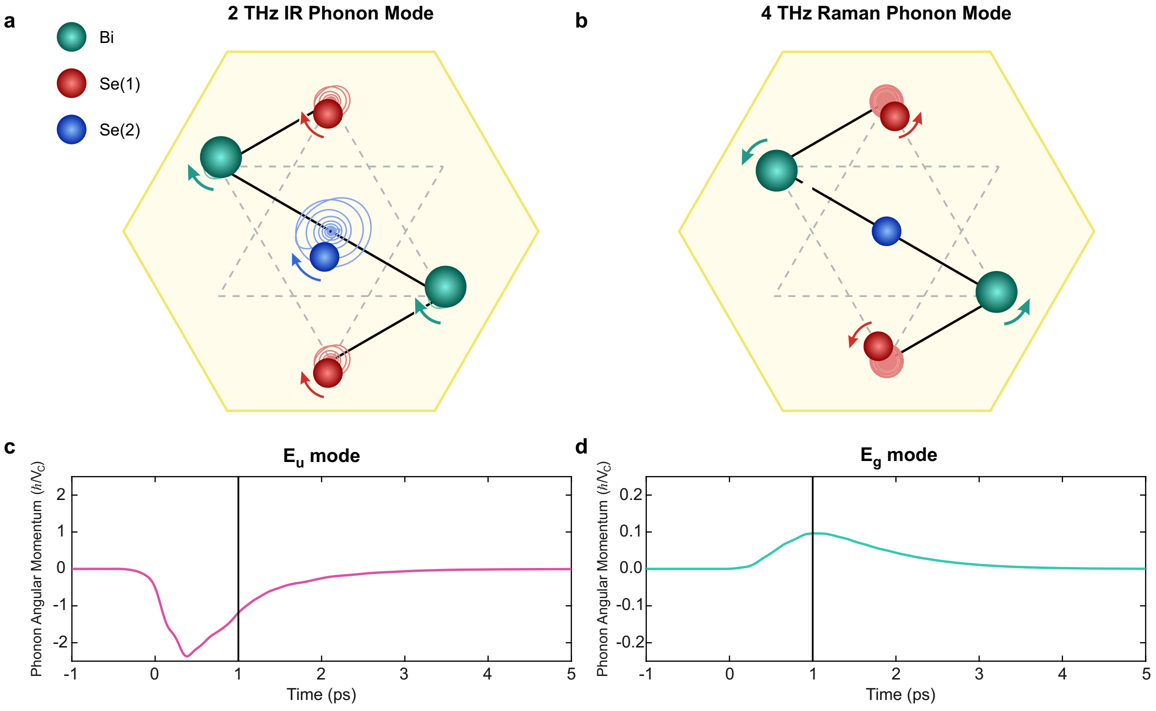

Fig. S3: **Computed real-space atomic trajectories. a-b**, Time-evolution of atomic displacements within the unit cell of Bi_2Se_3, obtained by solving the system of coupled equations of motion (Eqs.S15-S18) with the full form of the coupling potential V (Eq.13). The solution is projected onto the *ab-initio* eigenvectors shown in Fig. S2c,d. Phonon amplitudes are normalized relative to each other for clearer visibility. The resulting paths visualize the predicted helicity reversal between the driven E_u and the coupled E_g modes (see Supplementary Movie S3). **c-d**, Quantitative *ab-initio* modeling of angular momentum dynamics of E_u and E_g modes (see Fig.3).

the lateral ion motion shows a clear circular trajectory adding up to a **real net angular momentum** L_z^{IR} and L_z^{R} per unit cell, which we calculated (see details in Methods and Supplementary Text S2) and plotted in Fig. 3c. The resulting opposite sense of circular ion motion for the E_u and E_g mode can be also clearly seen, leading to opposite signs of the z-components (parallel to crystalline c-axis) of the respective angular momenta. In this sense the captions in Fig. 3c could have been misleading as the negative sign of L_z^{IR} was hidden in the normalization faction ("-0.041x"). We therefore changed all L to L_z and explicitly labelled "$-L_z^{IR}$".

In Supplementary Movie S3 and Figs. S3c,d, the time evolution clearly traces the delayed rise of the E_g mode's real angular momentum with opposite sign. This implies once again that a) **real angular momentum** from microscopic ion motion is **transferred** from one normal mode motion to the other and b) that this would violate angular momentum conservation in free space, but perfectly fulfills the **conservation of pseudo angular momentum** within the solid.

Therefore, following from 1), 2), and 3), our experiments demonstrate the first redistribution of angular momentum among lattice modes and is therefore **relevant** for angular momentum dissipation (e.g. after demagnetization) through the lattice.

Actions:

- We clarified explicitly the discussion of real vs. pseudo (or crystal) angular momentum throughout the **Main** manuscript. See e.g. **lines 46-49**, **lines 122-125**, **lines 179-182**, **lines 227-229**.
- We clarified the governing impact of the point group symmetry in **lines 201-202**.
- We added an extended symmetry analysis to the SI (**Supplementary Text S1**, **Figs. S1,S2**), which proves the helicity reversal when choosing a consistent right-handed basis.
- We added microscopic trajectories from DFT to SI (**Fig. S3** and **Supplementary Movie S3**), showing microscopic "real" angular momentum from the ion motion and helicity reversal between the anharmonically coupled modes.
- In **Fig. 3c**, we changed the naming of the "real" microscopic angular momentum z-components to L_z^{R} and $-L_z^{\mathrm{IR}}$ for a clearer understandability.
- Furthermore, we updated the **Methods** section on "First-principles calculations" to detail how quantitative L_z and the corresponding microscopic ion trajectories are obtained by using the experimentally measured THz field transients.

Angular momentum is not a conserved quantity in a solid. The only symmetry concept that matters is the irreducible representation of the crystal group. Helicity, that is, the projection of angular momentum along a chosen axis of rotation, can be an additional label when a single rotational

*axis exists, but **only** in such cases. For instance, in cubic crystals, with multiple non-collinear rotation axes, helicity is generally a useless label, and the irreducible representations of the full space group are the only proper classification. Moreover, even in the case of a single discrete axis, say, C_n, helicity is conserved only modulo n (what the authors note and refer to as Umklapp scattering). More importantly, other symmetry operations of the group will generally mix the helicity eigenvectors. Hence, helicity is not a symmetry-protected label, unlike in free space.*

Reply 3.B

We fully agree with Reviewer #3 on this most important aspect of our work: By translating concepts of angular momentum conservation from free space (continuous rotational symmetry) to a solid, everything is determined by the crystal's discrete symmetry group and thus helicity and angular momentum can be only be conserved modulo n in C_n! In our experiment, co-propagating pump- and probe-pulses define a unique optical axis at normal incidence to the to Bi_2Se_3 surface, thus coinciding with the crystalline c-axis (as confirmed by XRD, Fig. S6). Importantly, the exact frame of reference (and its handedness) is given by the two transversal light field components (E_x^{THz}, E_y^{THz}), which we directly measure with sign sensitivity at the sample position as previously detailed in Ref.[8].

Therefore, our experiment provides a consistent frame of reference and defines all involved helicity states with respect to a single axis of rotation, which coincides with the C_3 axis of the Bi_2Se_3 hexagonal unit cell. According to Streib[6], this lab frame defined by the external fields, is the reason why we can prepare and measure a real (spin) angular momentum of the excited phonon. For the first time in an experiment, we can therefore reliably confirm a helicity reversal between the driven IR-active phonon and an anharmonically coupled Raman-active phonon, representing the transfer of microscopic "real" angular momentum (see **Reply 3.1** above) between two coupled normal modes while conserving pseudo angular momentum. Please see below **Reply 3.2**, why we believe the rotational Umklapp picture is a good analogy to convey this fundamental process to a general audience.

Actions:

- Concerning **Symmetry discussion**: see Actions to **Reply 3.A** above.

- We prominently mention the limitations of the concept of angular momentum in a crystal with discrete rotational symmetry in the introduction of the **Main** manuscript (**lines 46-49**).
- We explain the common axis of reference for the THz electric field measurement ($E_x^{\mathrm{THz}}, E_y^{\mathrm{THz}}$) and phonon amplitude ($Q_\alpha^{\mathrm{R}}, Q_\beta^{\mathrm{R}}$), in the **Main** manuscript in **lines 101-103**.
- In **Fig. 1b** we added the c-axis and perpendicular ab-plane, and explicitly labeled the c-axis by its C_3 symmetry.
- In the **Methods** section on "Probing $\mathrm{E_g}$ phonon trajectories", we clarified the common symmetry of the measurements of the light polarization state ($E_x^{\mathrm{THz}}, E_y^{\mathrm{THz}}$) and phonon polarization state ($Q_\alpha^{\mathrm{R}}, Q_\beta^{\mathrm{R}}$).
- In the **Methods** section on "Helicity state characterization", we additionally mention the axis of reference coinciding with Bi_2Se_3's C_3 symmetry axis.

Concerning its symmetry properties, in Bi_2Se_3 we have the following: (i) The two circular components of the Raman mode, $Q_a \pm iQ_b$, or any other proper linear transformation, make an irreducible E_g representation. (ii) The in-plane components of the angular momentum, J_x and J_y, and the bilinear combinations $\{2J_xJ_y, J_x^2 - J_y^2\}$ also transform like E_g. (iii) The angular momentum transforms as $E_g + A_{2g}$. Consistently, the combinations $Q_x\dot{Q}_y - Q_y\dot{Q}_x$ and $Q_a\dot{Q}_b - Q_b\dot{Q}_a$, which the authors define as the z-component of ***L****, transform as the antisymmetric A_{2g}. This ends the point group discussion of the problem.*

Reply 3.C

We thank the reviewer for summarizing these symmetry insights on point. We believe that this point group discussion is in full agreement with our new symmetry analysis in **SI S1** and our **Reply 3.A** above.

Actions:

- We have added a comprehensive symmetry analysis as the **Supplemental Text S1**.

Despite these reservations, I am inclined to support the publication of the paper in Nature Physics, provided all the caveats noted above are very clearly addressed in the text. At the same time, it is essential that the limitations of the concept of angular momentum in a crystal are transparently acknowledged; failing to do so would undermine the credibility of the work. The authors should also address the following additional, less critical points.

Reply 3.D

We are deeply grateful for the reviewer's overall supportive evaluation, assessing a suitability for Nature Physics. We hope that the revised version addresses all concerns adequately. Of course, we also carefully addressed the following less critical comments, which were legitimately pointed out by Reviewer #3:

1) The references to magnetization are misleading, and their connection to the present work should be toned down. The Einstein–de Haas effect involves the conservation of real angular momentum, whereas this paper deals, at best, with a quasi-angular momentum.

Reply 3.1

The reviewer is absolutely right that our work neither directly deals with magnetization nor spin-lattice coupled phenomena. This choice is on purpose to focus on pure lattice angular momentum dynamics without having to rely on questionable magneto-optic signals as most of the experimental works in this field did so far. We have therefore toned down the references to magnetic phenomena in the revised manuscript.

Nevertheless, we still believe our findings have fundamental implications for magnetization or spin-lattice coupled phenomena. We agree that the original Einstein–de Haas effect concerns the conservation of real angular momentum between magnetization and macroscopic rotation of a finite sample. While we do not investigate this effect directly, angular momentum transfer is a key microscopic ingredient in the broader sequence of processes that can ultimately lead to a macroscopic rotation of the sample under finite boundary conditions. Our work focuses on one such microscopic step, the transfer of angular momentum between lattice vibrations through nonlinear (anharmonic) phonon interactions. This process provides a purely lattice-based pathway for angular momentum exchange. Although distinct from spin–lattice coupling, it becomes important after ultrafast

demagnetization, where a subset of strongly spin–lattice–coupled phonons initially receives the angular momentum from the electron spins[9,10]. This angular momentum needs to be redistributed and ultimately funneled into surface shear modes, as evidenced by the ultrafast Einstein–de Haas effect[11].

Actions:

- We partly toned down the references to magnetic phenomena across the whole manuscript.
- We also now explicitly mention the "delicate" interpretation of magneto-optic phonon signals and refer to the recent work by Merlin[12] (**line 64** in the **Main** manuscript).
- We also clarified the relation to the Einstein-de Haas effect by more specifically referring to the "ultrafast" Einstein-de Haas and "ultrafast demagnetization sequences", where required (e.g. **lines 71**, **240**).

2) The analogy with the Umklapp processes considered by Bloembergen (Ref. [32]) is similarly misleading. In that work, Umklapp refers to the angular momentum taking up by the sample as a whole. The incident and transmitted circularly polarized photons carry a well-defined spin angular momentum, which is "real," in contrast to the helicity defined in a solid-state context.

Reply 3.2

We thank the reviewer for pointing out this lack of clarity in the previous version of our manuscript. Indeed, our **Reply 3.A** above details how "real" angular momentum is first transferred from the light field to the E_u phonon (via absorption) and then further to the E_g phonon (via lattice anharmonicity). The latter we refer to an Umklapp process. Here, we briefly elaborate on the analogy to established Umklapp processes, which is twofold:

a) Analogy "photon → phonon": The "photonic" rotational Umklapp scattering by Bloembergen[13] describes the angular momentum conservation in nonlinear optics for "2 photons in → 1 photon out", e.g. in the case of second harmonic generation $\omega + \omega \rightarrow 2\omega$. The reviewer is right; in this case, the observed helicity reversal corresponds to a real change in spin angular momentum. Nevertheless, translating the concept to nonlinear phononics ("2 phonons in → 1 phonon out"),

in our case $\Omega+\Omega \rightarrow 2\Omega$, leads to a helicity reversal based on the same symmetry considerations detailed by Bloembergen [13].

b) Analogy "pseudo linear momentum $\rightarrow$ pseudo angular momentum": Three-phonon Umklapp scattering, being a fundamental concept of solid state physics [14], exactly describes the pseudo linear momentum conservation dictated by a discrete translational invariance of a solid. Translating this concept to discrete rotational invariance leads to pseudo angular momentum conservation and exactly describes the central result of this work.

Due to the striking consistency with these two established concepts, we experienced in numerous scientific discussion and presentations that the term "rotational phonon-phonon Umklapp scattering" conveys exactly the main message of this work and facilitates the understandability for a broader audience familiar with nonlinear optics and fundamentals of solid-state physics.

Actions:

- We clarified the analogy to the conservation laws in nonlinear optics and to the established linear momentum phonon-phonon Umklapp process in **lines 231-234** of the **Main** manuscript.

3) D3d is an achiral group. Hence, chiral ≠ axial. The authors should refrain from using the term 'chiral' to refer to axial modes. This point is generally discussed in a recent paper by Merlin [PNAS Nexus 4 (2025)].

Reply 3.3

We strongly support using the notion of "chiral phonons" carefully and accurately. Therefore, already in the previous version of the manuscripts, we tried to refrain actively from the term "chiral" phonons, despite contemporary interpretations arguing with a non-zero linear momentum of the excitation pulse. In the revised version therefore, we unambiguously stick to the strict definition of zone center circularly polarized phonons, i.e. axial phonons, according to our recent taxonomy in Ref. [15].

Actions:

- We have replaced misleading wordings concerning chiral phonons throughout the whole manuscript and abstract.

- We additionally refer to the paper by Merlin[12] to improve the general awareness on that topic (**line 57**, **Main** manuscript).

4) The authors should not use Q_x and Q_y to refer to the Raman phonon components since they are not components of a vector. Something like Q_α and Q_β would be fine.

Reply 3.4

Even though the components of the E_g mode relate to two orthogonal ionic motions at the involved lattice sites, they do not necessarily correspond to the $(E_x^{\mathrm{THz}}, E_y^{\mathrm{THz}})$ frame of reference. We thus agree with the reviewer that the x, y indices of the Raman mode are misleading.

Actions:

- We have replaced the notation Q_x^{R} and Q_y^{R} with Q_α^{R} and Q_β^{R} throughout the whole manuscript.

5) The IR mode at 2 THz was first identified by LaForge at al. [PRB 81, 125120 (2010)]. A reference to this paper should be given.

Reply 3.5

We thank the reviewer for pointing out this missing reference.

Actions:

- We have added the reference to LaForge et al.[16] in the discussion of the 2 THz IR-active mode in **lines 81** and **318** in the **Main** text and **Methods** section, respectively.

References

[1] Juraschek, D. M. & Maehrlein, S. F. Sum-frequency ionic raman scattering. *Physical Review B* **97**, 174302 (2018).

[2] Johnson, C. L., Knighton, B. E. & Johnson, J. A. Distinguishing nonlinear terahertz excitation pathways with two-dimensional spectroscopy. *Physical Review Letters* **122**, 073901 (2019).

[3] Luo, J. *et al.* Large effective magnetic fields from chiral phonons in rare-earth halides. *Science* **382**, 698–702 (2023).

[4] Basini, M. *et al.* Terahertz electric-field-driven dynamical multiferroicity in SrTiO3. *Nature* **628**, 534–539 (2024).

[5] Biggs, M. F. *et al.* Ultrafast faraday rotation probe of chiral phonon-polaritons in LiNbO3 (2025). ArXiv:2507.22232.

[6] Streib, S. Difference between angular momentum and pseudoangular momentum. *Physical Review B* **103**, L100409 (2021).

[7] Wefers, M. M., Kawashima, H. & Nelson, K. A. Optical control over two-dimensional lattice vibrational trajectories in crystalline quartz. *The Journal of Chemical Physics* **108**, 10248–10255 (1998).

[8] Frenzel, M. *et al.* Quartz as an accurate high-field low-cost thz helicity detector. *Optica* **11**, 362–370 (2024).

[9] Tauchert, S. R. *et al.* Polarized phonons carry angular momentum in ultrafast demagnetization. *Nature* **602**, 73–77 (2022).

[10] Mrudul, M. S., Weißenhofer, M. & Oppeneer, P. M. Generation of phonons with angular momentum during ultrafast demagnetization. *Physical Review B* **112**, L180407 (2025).

[11] Dornes, C. *et al.* The ultrafast einstein-de haas effect. *Nature* **565**, 209–212 (2019).

[12] Merlin, R. Magnetophononics and the chiral phonon misnomer. *PNAS Nexus* **4**, pgaf002 (2025).

[13] Bloembergen, N. Conservation laws in nonlinear optics. *Journal of the Optical Society of America* **70**, 1429–1436 (1980).

[14] Kittel, C. *Introduction to Solid State Physics* (John Wiley & Sons, Inc., New York, 2005), 8th edn.

[15] Juraschek, D. M. *et al.* Chiral phonons - perspective. *under review* (2025).

[16] LaForge, A. D. *et al.* Optical characterization of Bi2Se3 in magnetic field: Infrared evidence for magnetoelectric coupling in a topological insulator material. *Physical Review B* **81**, 125120 (2010).

The mathematics underlying the results in this paper is fairly simple. For the anharmonic interaction $V = V_0\left[(Q_x^2 - Q_y^2)Q_\alpha + 2Q_xQ_yQ_\beta\right]$, the equations of motion are

$$\ddot{Q}_\alpha + \Omega_R^2 Q_\alpha = -V_0(Q_x^2 - Q_y^2) \qquad \ddot{Q}_\beta + \Omega_R^2 Q_\beta = -2V_0 Q_x Q_y \quad .$$

Here, the two components of the polar and Raman modes are labeled (Q_x, Q_y) and (Q_α, Q_β), respectively. For $Q_x = A\sin\omega t$ and $Q_y = A\cos\omega t$, we get

$$Q_\alpha = +\frac{A^2V_0}{\Omega_R^2 - 4\omega^2}\cos 2\omega t \qquad Q_\beta = -\frac{A^2V_0}{\Omega_R^2 - 4\omega^2}\sin 2\omega t \quad .$$

Thus, if the coherent E_u mode is, say, left handed, the coherently driven E_g mode is right handed and vice versa. The experimental results are in very good agreement with this analysis, and the evidence that the driving mechanism for the E_g phonon is lattice anharmonicity (rather than electron-phonon interaction) is strong. The remaining question is: what, if anything, does this have to do with angular momentum transfer, as claimed by the authors?

Angular momentum is not a conserved quantity in a solid. The only symmetry concept that matters is the irreducible representation of the crystal group. Helicity, that is, the projection of angular momentum along a chosen axis of rotation, can be an additional label when a single rotational axis exists, but **only** in such cases. For instance, in cubic crystals, with multiple non-collinear rotation axes, helicity is generally a useless label, and the irreducible representations of the full space group are the only proper classification. Moreover, even in the case of a single discrete axis, say, C_n, helicity is conserved only modulo *n* (what the authors note and refer to as Umklapp scattering). More importantly, other symmetry operations of the group will generally mix the helicity eigenvectors. Hence, helicity is not a symmetry-protected label, unlike in free space.

Concerning its symmetry properties, in Bi2Se3 we have the following: (*i*) The two circular components of the Raman mode, $Q_a \pm iQ_b$, or any other proper linear transformation, make an irreducible E_g representation. (*ii*) The in-plane components of the angular momentum, J_x and J_y, and the bilinear combinations $\{2J_xJ_y, J_x^2 - J_y^2\}$ also transform like E_g. (*iii*) The angular momentum

transforms as $E_g + A_{2g}$. Consistently, the combinations $Q_x\dot{Q}_y - Q_y\dot{Q}_x$ and $Q_a\dot{Q}_b - Q_b\dot{Q}_a$, which the authors define as the *z*-component of **L**, transform as the antisymmetric A_{2g}. This ends the point group discussion of the problem.

Despite these reservations, I am inclined to support the publication of the paper in Nature Physics, provided all the caveats noted above are very clearly addressed in the text. At the same time, it is essential that the limitations of the concept of angular momentum in a crystal are transparently acknowledged; failing to do so would undermine the credibility of the work. The authors should also address the following additional, less critical points:

1. The references to magnetization are misleading, and their connection to the present work should be toned down. The Einstein–de Haas effect involves the conservation of real angular momentum, whereas this paper deals, at best, with a quasi-angular momentum.
2. The analogy with the Umklapp processes considered by Bloembergen (Ref. [32]) is similarly misleading. In that work, Umklapp refers to the angular momentum taking up by the sample as a whole. The incident and transmitted circularly polarized photons carry a well-defined spin angular momentum, which is "real," in contrast to the helicity defined in a solid-state context.
3. D_{3d} is an achiral group. Hence, chiral ≠ axial. The authors should refrain from using the term 'chiral' to refer to axial modes. This point is generally discussed in a recent paper by Merlin [PNAS Nexus 4 (2025)].
4. The authors should not use Q_x and Q_y to refer to the Raman phonon components since they are not components of a vector. Something like Q_a and Q_b would be fine.
5. The IR mode at 2 THz was first identified by LaForge at al. [PRB 81, 125120 (2010)]. A reference to this paper should be given.
